# The CDR1 and Other Regions of Immunoglobulin Light Chains are Hot Spots for Amyloid Aggregation

**DOI:** 10.1038/s41598-019-39781-3

**Published:** 2019-02-28

**Authors:** Robin Axel Ruiz-Zamora, Simon Guillaumé, Youssra K. Al-Hilaly, Zahraa Al-Garawi, Francisco Javier Rodríguez-Alvarez, Guadalupe Zavala-Padilla, Julio I. Pérez-Carreón, Sandra L. Rodríguez-Ambriz, Guillermo A. Herrera, Baltazar Becerril-Luján, Adrián Ochoa-Leyva, Jorge Melendez-Zajgla, Louise Serpell, Luis del Pozo-Yauner

**Affiliations:** 1 Instituto Nacional de Medicina Genómica, Periférico Sur No. 4809, Col. Arenal Tepepan, Delegación Tlalpan, Ciudad de México, 14610 Mexico; 2 School of Life Sciences, University of Sussex, Falmer, Brighton, East Sussex BN1 9QG United Kingdom; 3 Chemistry Department, College of Science, Mustansiriyah University, Baghdad, Iraq; 4 Instituto de Biotecnología, Universidad Nacional Autónoma de México, Avenida Universidad 2001, Colonia Chamilpa, Cuernavaca, 62210 Morelos Mexico; 5 Centro de Desarrollo de Productos Bióticos, Instituto Politécnico Nacional, Carr. Yautepec-Jojutla Km 6, Calle CEPROBI No. 8, Col. San Isidro, Yautepec, 62731 Morelos Mexico; 6 Department of Pathology and Translational Pathobiology, Louisiana State University Health Sciences Center Shreveport, 1501 Kings Hwy, Shreveport, LA 71103 USA

## Abstract

Immunoglobulin light chain-derived (AL) amyloidosis is a debilitating disease without known cure. Almost nothing is known about the structural factors driving the amyloidogenesis of the light chains. This study aimed to identify the fibrillogenic hotspots of the model protein 6aJL2 and in pursuing this goal, two complementary approaches were applied. One of them was based on several web-based computational tools optimized to predict fibrillogenic/aggregation-prone sequences based on different structural and biophysical properties of the polypeptide chain. Then, the predictions were confirmed with an *ad-hoc* synthetic peptide library. In the second approach, 6aJL2 protein was proteolyzed with trypsin, and the products incubated in aggregation-promoting conditions. Then, the aggregation-prone fragments were identified by combining standard proteomic methods, and the results validated with a set of synthetic peptides with the sequence of the tryptic fragments. Both strategies coincided to identify a fibrillogenic hotspot located at the CDR1 and β-strand C of the protein, which was confirmed by scanning proline mutagenesis analysis. However, only the proteolysis-based strategy revealed additional fibrillogenic hotspots in two other regions of the protein. It was shown that a fibrillogenic hotspot associated to the CDR1 is also encoded by several κ and λ germline variable domain gene segments. Some parts of this study have been included in the chapter “*The Structural Determinants of the Immunoglobulin Light Chain Amyloid Aggregation*”, published in Physical Biology of Proteins and Peptides, Springer 2015 (ISBN 978-3-319-21687-4).

## Introduction

Immunoglobulin light chain (AL) amyloidosis is a serious disease characterized by the systemic deposition, in the extracellular compartment, of a monoclonal free light chain as insoluble fibrils^1^. Despite the progress achieved in the last two decades in the therapeutic management of AL-amyloidosis^2,3^, it remains as an incurable and often fatal disease. To some extent, this is due to the insufficient understanding regarding the structural factors that drive the amyloidogenesis of the light chains. This has halted the development of more effective therapeutic approaches aiming to suppress the key molecular event of the disease pathogenesis: the misfolding of the light chain and its subsequent self-assembly into the ordered amyloid fibrils^4,5^. However, misfolding can also result in light chains forming a non-fibrillar structure^6–8^. Since the specific microscopic ordering adopted by the light chain in the tissue deposits has a strong influence on the clinical course of the disease^9^, there is much interest in understanding what makes a light chain amyloid-prone. There is an increasing body of evidence indicating that the ability of a protein to form amyloid fibrils is contained in short stretches of its sequence, known as fibrillogenic hotspots^10^. As a distinctive property, these protein segments can autonomously form fibrillar aggregates with characteristics of amyloid^11^. Moreover, it has been shown that the aggregation of the full-length amyloid precursor can be modulated by targeting their fibrillogenic region(s) with site-directed mutagenesis or by the binding with specific ligands^12,13^. Correspondingly, a naturally non-amyloidogenic protein can be converted into an amyloid-prone one by inserting into its sequence non-destabilizing short amyloid-forming segments taken from an amyloidogenic polypeptide^10^. X-ray diffraction studies of microcrystals formed by fibrillogenic peptides have revealed that they can form a variety of cross β spines, composed of β-sheets running along the longitudinal axis of the crystal lattice, with the amino acid side chains interdigitated in a complementary dry “steric zipper”^14,15^. It is thought that the steric zipper is the structural motif characterizing the amyloid fibril core. Information regarding the characteristics of the fibrillogenic sequences of the light chains is very scarce. The identification of such sequences is critical for understanding the structural basis of amyloidogenesis of the light chains. Moreover, this could foster the development of better therapeutic and/or diagnostic strategies in AL amyloidosis, since the pro-fibrillogenic hot-spots represent potential targets for theranostic agents^16^. Thus, this study aimed to identify the fibrillogenic hotspots of the model protein 6aJL2^17^, a recombinant (r) light chain variable domain (V_L_) encoded by the λ6 germline gene segment *IGLV6-57* (6a). Previous studies have shown that the light chains encoded by *IGLV6-57*, the only gene segment composing the λ6 subgroup, display a high propensity to aggregate as amyloid *in vivo*^18–22^. With only one clinically proven exception^23^, all monoclonal λ6 light chains identified so far has been shown to be involved in amyloid deposition^22^. Moreover, we have shown that 6aJL2 can form amyloid-like fibrils in physiological relevant conditions of pH, temperature, and ionic strength^24^. These findings suggest that as yet unidentified amyloidogenic sequences encoded in the germline *IGLV6-57* could be part of the structural factors that make λ6 light chains highly prone to aggregate as amyloid fibrils^17,24,25^. Being a protein with germline sequence, 6aJL2 is an ideal model to study the contribution of germline-encoded fibrilogenic sequences to the propensity of the λ6 light chains to aggregate as amyloid. For this purpose, an experimental strategy composed by two complementary approaches was applied (Fig. S1). In one approach, the sequence of 6aJL2 protein was scanned with two web-based computational tools, ZypperDB^26,27^ (http://services.mbi.ucla.edu/zipperdb/) and AmylPred2^28^ (http://aias.biol.uoa.gr/AMYLPRED2/), optimized to predict fibrillogenic/aggregation-prone sequences based on different structural and biophysical properties of the polypeptide chain. Then, the predictions were confirmed with an *ad-hoc* synthetic peptide library. In the second approach, protein 6aJL2 was proteolyzed with trypsin, and the products were incubated in aggregation-promoting conditions. Then, the aggregation-prone fragments were identified by combining standard proteomic methods, and the results validated by analysing the aggregation behaviour of a set of synthetic peptides with the sequence of the tryptic fragments. Both strategies coincided to identify a fibrillogenic hotspot located at the CDR1 and β-strand C of the protein, which was confirmed by scanning proline mutagenesis analysis. However, only the proteolysis-based strategy revealed additional fibrillogenic hotspots in two other regions of the protein. Furthermore, we show that a fibrillogenic hotspot centre within the CDR1 is also encoded by other germline gene segments belonging to different κ and λ V_L_ subgroup, indicating the general applicability of our findings for AL.

## Results

### Computational prediction of fibrillogenic/aggregation-prone sequences

**Figure 1:**
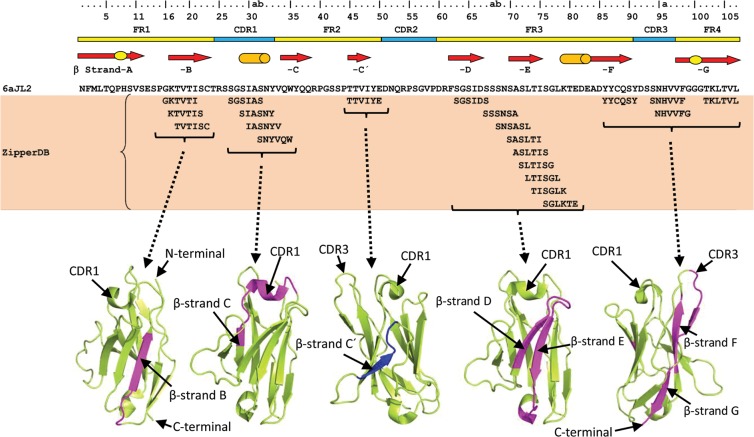
Segments of 6aJL2 protein predicted to be fibrillogenic by the computational tool ZipperDB^26,29^ (https://services.mbi.ucla.edu/zipperdb/). The hexamers shown are those with a Rosetta energy ≤−23 kcal/mol, which are predicted to form fibrils^27^. The location of the clusters of the amyloidogenic hexamers is shown highlighted in magenta in the three-dimensional structure of 6aJL2 protein (bottom). The regions of 6aJL2 protein with β-strands or helix conformation in the native state are indicated by arrows and cylinders, respectively (Top). The oval figures in the first and last arrows represent, respectively, the sheet-switch motif characterizing the structure of the N-terminal segment, and the β-bulge centred at Gly100 in the β-strand G. The residue numbering and the location of the CDR/FR regions are according to *Chothia and Lesk*^69^. The graphical representations of 6aJL2 structure were prepared with PyMOL^70^, based on the structure contained in the PDB 2W0K.

**Figure 2:**
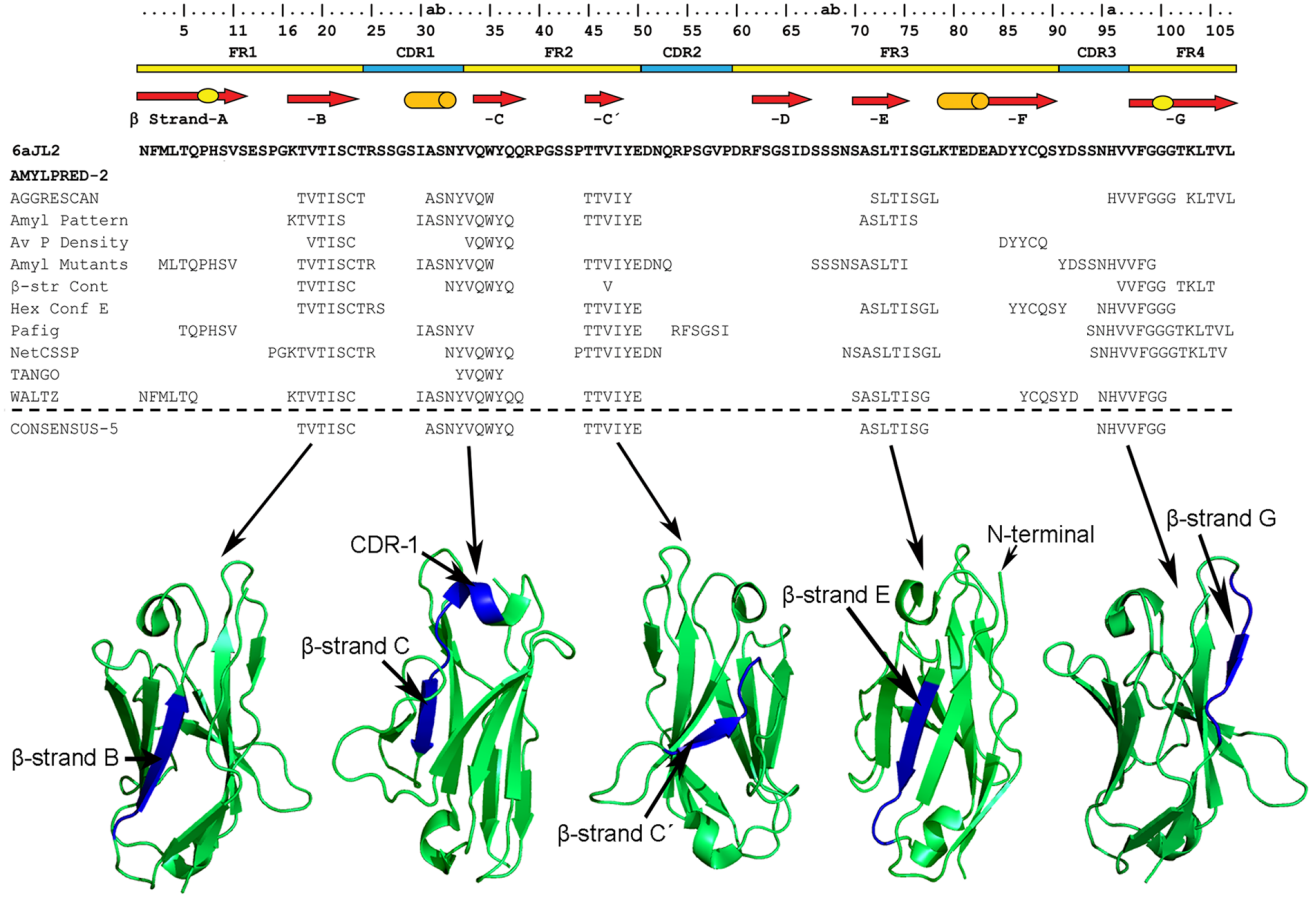
Consensus sequences (CONSENSUS-5) generated by the AmylPred2 tool from the analysis of the 6aJL2 protein with 10 different algorithms designed for predicting aggregation/fibrillogenic-prone sequences^28^. The individual prediction of each method is also shown. The regions of 6aJL2 protein with β-strands or helix conformation in the native state are indicated as in Fig. 1. The residue numbering and the location of the CDR/FR regions are according to *Chothia and Lesk*^69^. The graphical representations of 6aJL2 structure were prepared with PyMOL^70^, based on the structure contained in the PDB 2W0K.

ZipperDB identified 21 hexamers in the 6aJL2 protein forming steric zippers with a fit energy calculated with Rosetta of −23 kcal/mol or lower. Segments with Rosetta energy equal to or below −23 kcal/mol are deemed to have high fibrillation propensity^27^ (Fig. 1). These hexamers cluster in four regions of the domain, the β-strand B, the Complementary Determining Region 1 (CDR1), the region spanning the β strands D and E, and that spanning the β-strand F, CDR3 and β-strand G (Fig. 1)^29^. AmylPred2 identified five fibrillogenic/aggregation-prone consensus sequences, which overlap with those identified by ZipperDB (Fig. 2)^29^.

### Aggregation assay of the prediction-based synthetic peptides

**Figure 3:**
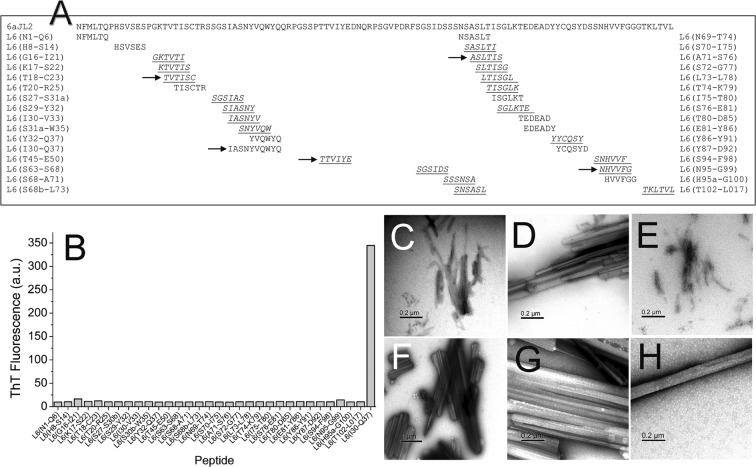
(**A**) Prediction-based synthetic peptide library composed of thirty-one hexapeptide and one decapeptide designed for testing the predictions of fibrillogenic/aggregation prone sequences in the rV_L_ protein 6aJL2^29^ generated by the web-based computational tools ZipperDB^26^ and AmylPred2^28^. The arrows point the consensus sequences predicted to be aggregation/fibrillogenic-prone by the tool AmylPred2. The sequences underlined and *italized* represent the hexapeptides forming steric zippers with a fit energy of −23 kcal/mol or lower, as calculated with RosettaDesign^71^. Segments with energies equal to or below this threshold are deemed to have high fibrillation propensity^27^. (**B**) Aggregation assay of the synthetic peptides composing the prediction-based peptide library. The data represented is the thioflavin T (ThT) fluorescence intensity of the peptide samples (250 µM peptide dilution in PBS pH 7.4 plus 0.05% Na Azide), measured after 24 hours of incubation at 37 °C with constant agitation. Two replicas of the experiment were performed with very similar results (Result not shown). (**C**–**H**) Transmission electron micrographs of the aggregates present in the end-point samples of the synthetic peptides Ile30-Gln37 (**C**,**D**), Ile30-Val33 (**E**), and Ser30b-Trp35 (**F**–**H**).

Based on the predictions generated by the tools ZipperDB and AmylPred2, a synthetic peptide library, composed by 31 hexapeptides and one decapeptide, was designed (Fig. 3A and Table S2). The library was composed by 21 hexamers with Rosetta energy of −23 kcal/mol or lower, and 10 hexamers with Rosetta energy higher than −23 kcal/mol, predicted by ZipperDB to have low propensity to form amyloid^27^. The decapeptide _30_IASNYVQWYQ_37_ was included, as it was recognized as potentially fibrillogenic by four of ten predictors consulted by the algorithm AmylPred2^28^. The aggregation behaviour of the synthetic peptides composing the prediction-based peptide library was assayed as described in Methods. After 24 hours of incubation, only the samples of the decamer Ile30-Gln37 and the hexamers Ile30-Val33 and Ser30b-Trp35 displayed cloudy appearance suggesting the presence of insoluble aggregates, but only those formed by Ile30-Gln37 displayed fluorescence in presence of ThT (Fig. 3B)^29^.

### Transmission Electron microscopy (TEM)

Peptide Ile30-Gln37 formed fibrils that associate in bundles, as well as large and irregular aggregates with crystalline morphology (Fig. 3C,D). Also, long and straight rod-like aggregates were observed, some of them associated in pairs (Fig. S3A). The hexapeptide Ile30-Val33 formed fibrils (Fig. 3E), while Ser30b-Trp35 formed paracrystalline aggregates of different morphology. Some were irregular (Fig. 3F), others were needle-like (Fig. S3C) or large and faceted (Fig. 3G). Long rod-like fibrous crystals were also observed (Fig. 3H and S3D). It is worth mentioning that both hexapeptides, Ile30-Val33 (_30_IASNYV_33_) and Ser30b-Trp35 (_30b_SNYVQW_35_), are contained within the sequence of Ile30-Gln37 (_30_IASNYVQWYQ_37_). No needle-like fibrous crystals or fibrils were observed in the remaining samples.

### Far-UV Circular Dichroism Spectroscopy

**Figure 4:**
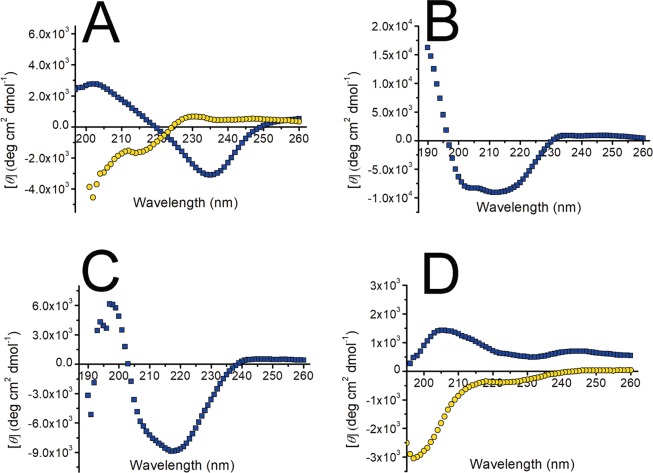
Far-UV circular dichroism spectra of peptide (**A**) Ile30-Gln37, (**B**) Ser26-Arg39, (**C**) tryptic fragment Thr18-Arg25-S-S-Thr80-Lys103, and (**D**) Phe62-Lys79 before (yellow circles) and after (blue squares) the fibrillogenesis assay, performed as described in Methods.

Despite having shown ThT fluorescence and fibrillar morphology under TEM, the Far-UV CD spectrum of the end-point aggregates of peptide Ile30-Gln37 was not typical of β conformation (Fig. 4A). Instead, it featured a band of negative ellipticity centred at 235 nm, and a second one of positive ellipticity with a maximum around 203 nm, a pattern probably determined by the aromatic moiety of the Tyr residues present in the peptide. Far-UV CD spectra characterized by one or more minima and maxima at different wavelength were also displayed by the hexapeptides with aromatic residues, in contrast to those with no aromatic residues, which displayed spectra characteristic of an unstructured polypeptide, both before and at the end of the incubation (Fig. S4).

### XRFD analysis of aggregates formed by synthetic peptides

**Figure 5:**
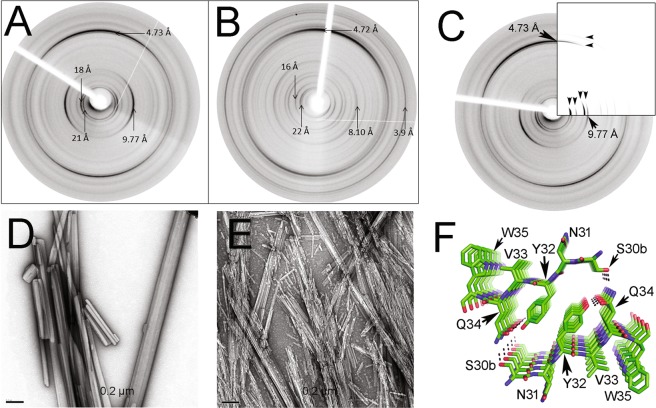
X-ray fibre diffraction (XRFD) patterns from partially aligned fibrils formed by synthetic peptides (**A**) Ser30b-Trp35 and (**B**) Ile30-Gln37 incubated in MilliQ-quality water plus Na azide 0.05%, as described in Methods. (**D**,**E**) show transmission electron micrographs of the aggregates whose XRFD pattern are shown in panels A and B, respectively. The images were obtained with a HITACHI-7100 transmission electron microscope (**C**). Comparison of the experimental XRFD pattern of the aggregates of the peptide Ser30b-Trp35, as shown in (A), and the simulated pattern (inserted quadrant) calculated based on the theoretical model shown in (**F**). Black arrows are shown to highlight positions of matching major diffraction signal positions on the equator and meridian. (**F**) Structural model of the steric zipper of peptide Ser30b-Trp35, generated by the computational tool ZipperDB^26^. The amino acid side chains are shown in stick representation and named with the one-letter code. The dotted lines represent intra- and interpeptide H-bonds stabilizing the crystal lattice. Figure prepared with PyMOL^70^.

To produce material suitable for X-ray fiber diffraction (XRFD) analysis, the peptides Ile30-Val33, Ser30b-Trp35, and Ile30-Gln37 were aggregated in the same conditions of temperature and speed of agitation used in the fibrillogenesis assays, but, instead of dissolving in PBS pH 7.4, the peptides were solubilized in milli-Q water plus 0.05% Na azide. Peptide Ile30-Val33 formed needle-like aggregates (Fig. S5A–C), while Ser30b-Trp35 formed rod-like fibres, large cylindrical aggregates of variable diameter and length, and irregular paracrystalline aggregates similar to those formed in PBS pH 7.4 (Fig. 5D and S5D–F). Ile30-Gln37 formed abundant aggregates with the appearance of long rigid fibrils, as well as thin needles (Fig. 5E and S5G–I). XRFD patterns from partially aligned aggregates of peptides Ser30b-Trp35 and Ile30-Gln37 showed the major reflections expected for amyloid^30,31^, with a strong diffraction signal on the meridian (vertical) at 4.7 Å (Fig. 5A,B). The position of all diffraction signals is shown in Table S6. Each pattern showed a series of very sharp equatorial diffraction signals arising from the crystalline morphology of the fibrillar structures. Ser30b-Trp35 gave rise to a sharp and strong diffraction signal at 9.77 Å which is commensurate with a β-sheet spacing for a steric zipper arrangement (Fig. 5A). The diffraction pattern for Ile30-Gln37 showed several equatorial signals with similar intensities at 12.4 Å, 11 Å and 8.1 Å (Fig. 5B and Table S6). Both diffraction patterns showed low angle diffraction signals at 21 Å and 18.2 Å for Ser30b-Trp35 and 22 Å for Ile30-Gln37 (Table S6). Ser30b-Trp35 equatorial diffraction signal at 18.2 Å may arise from the arrangement of two β-strands separated by 9.77 Å oriented antiparallel to one another. 21 Å is approximately the chain length for a hexapeptide (3.5 Å x6). The structural model for the steric zipper of the peptide Ser30b-Trp35, generated by the computational tool ZipperDB, was arranged into a unit cell with dimensions 21 Å × 18.2 Å × 4.73 Å, α = β = γ = 90° and the diffraction pattern was calculated to enable the model structure to be compared to the experimental data (Fig. 5C,F). Several major diffraction signal positions, on the equator as well as meridian, match well between the calculated and experimental XRFD patterns. The diffraction pattern from Ile30-Gln37 gives the lowest visible and measurable angle signal at 22 Å. However, careful examination of the pattern reveals a lower angle diffraction peak, very close to the back stop which may suggest a lower angle signal is present. The length of an extended β-strand with 10 amino acid residues is approximately 33 Å, which may align well with the weak low angle equatorial signal. Alternatively, the 10 residues peptide may form a bend similar to previous structures published for amyloid peptides^32,33^. The diffraction signals at 22 Å and 11 Å may be related and the 11 Å may represent the sheet spacing which would accommodate the large and bulky side chains. A unit cell *a* = 32 Å, *b* = 22 Å, *c* = 4.72 Å, α = β = γ = 90° could explain the observations at 8.1 Å [4 0 0], 16 Å [2 0 0] and 11 Å [0 2 0].

### Proteolysis of 6aJL2 protein with trypsin

**Figure 6:**
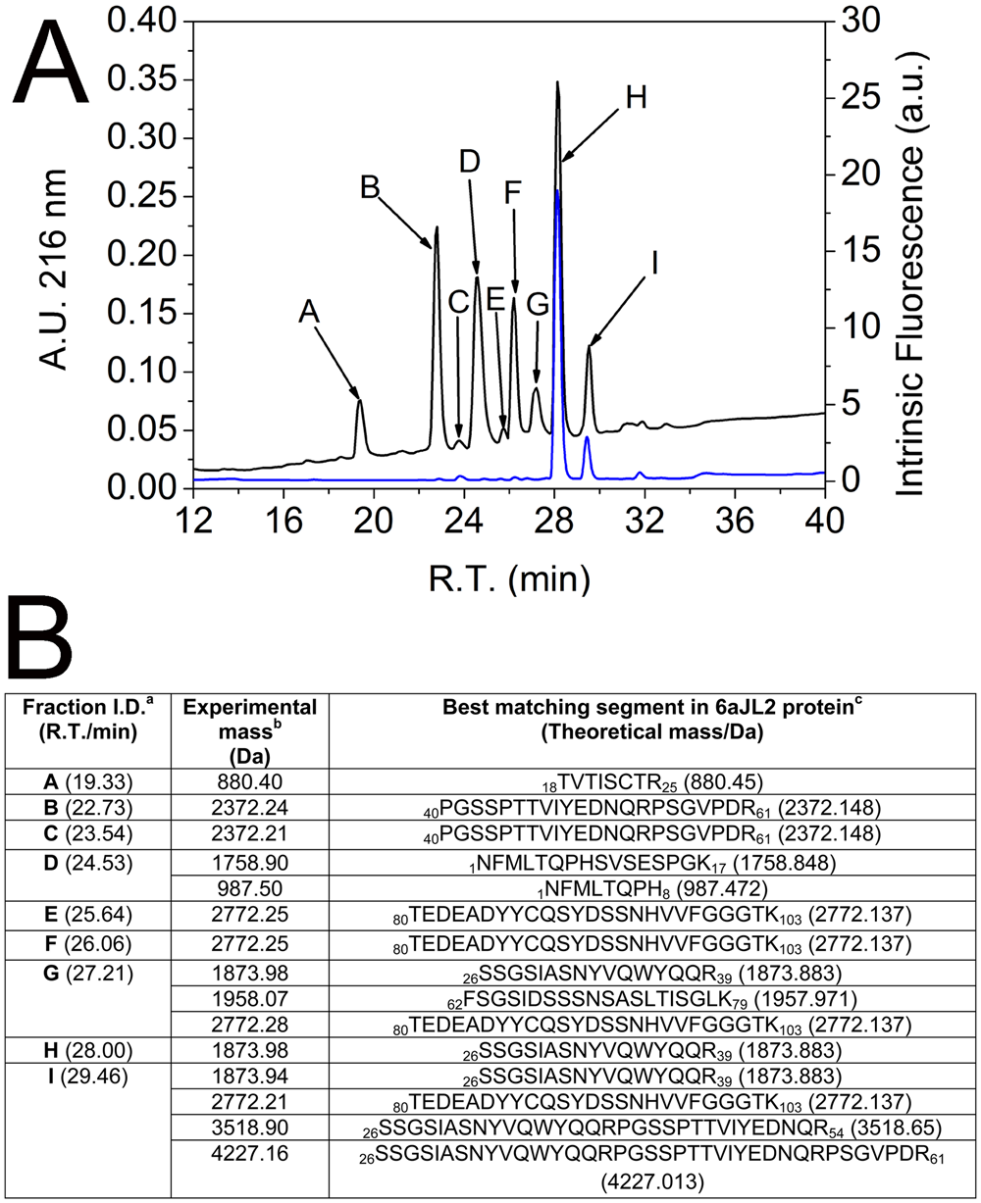
(**A**) RP-HPLC elution profile of the products of the proteolysis of soluble 6aJL2 protein with trypsin. Prior to be injected into the column, the sample was reduced by adding 10 mM dithiothreitol (DTT). The data represented are the time-dependence variation of (black line) absorbance at 216 nm or (blue line) intrinsic fluorescence at 350 nm, exciting the sample at 295 nm. (**B**) Identification of the proteolytic fragments of 6aJL2 protein contained in the chromatography fractions collected in A) by MALDI-TOF mass spectrometry analysis. ^***a***^refers to the fractions identified with the same letter code in panel (A). R.T./min refers to the retention time (minutes) of each fraction in the RP-HPLC analysis. ^***b***^refers to the mass (Da) determined experimentally by MALDI-TOF mass spectrometry analysis. ^***c***^refers to the segment of protein 6aJL2 identified as the best-matching sequence for each experimental mass determined by means of the web-based computational tool FindPept (http://web.expasy.org/findpept/).

The increasing knowledge about the amino acid sequences conferring propensity to form amyloid have stimulated the development of several computational algorithms optimized to recognize such aggregation hot-spots^34^. Most of these methods rely solely on the sequence analysis, thus they are relatively ineffective for identifying aggregation-prone sites that may be non-contiguous in sequence; buried inside the native structure, or dependent on structural factors restricting the conformational freedom in the context of a globular protein, such as disulphide bonds^35^. Based on this argument, a second experimental approach, based on limited proteolysis of 6aJL2 protein was applied. This approach is based on the observation that bulky and charged residues, as well as proline, tend to be positioned at the flanks of aggregation-prone regions in globular proteins, a pattern that is believed to be evolutionarily selected to control protein aggregation^36,37^. In agreement with this observation, it was found that the regions of 6aJL2 predicted as fibrillogenic by the web-based tool AmylPred2 are flanked by Arg or Lys residues (Fig. S7). As trypsin cleaves the peptide backbone at the C-terminal side of Arg and Lys residues, we hypothesized that this protease can be used for releasing fragments of 6aJL2 protein bearing aggregation-promoting sequences whose aggregation depends on driving forces not considered by the computational tools used. In two (Arg39 and Arg54) of the seven trypsin cleavage sites distributed along the sequence of 6aJL2 protein, a proline residue occupies the C-terminal contiguous position (Fig. S8), which is predicted to severely decrease the cleavage probability (http://web.expasy.org/peptide_cutter/). However, fragments generated by cleavage at these sites appeared early during the proteolysis of the native protein (Fig. S9). The RP-HPLC profile of the end-point products of the proteolysis of 6aJL2 protein (described in Method), as well as the identity of each proteolytic fragment, as determined by MALDI-TOF mass spectrometry (MS) analysis, are shown in Fig. 6.

### Fibrillogenesis of the tryptic fragments of 6aJL2 protein

**Figure 7:**
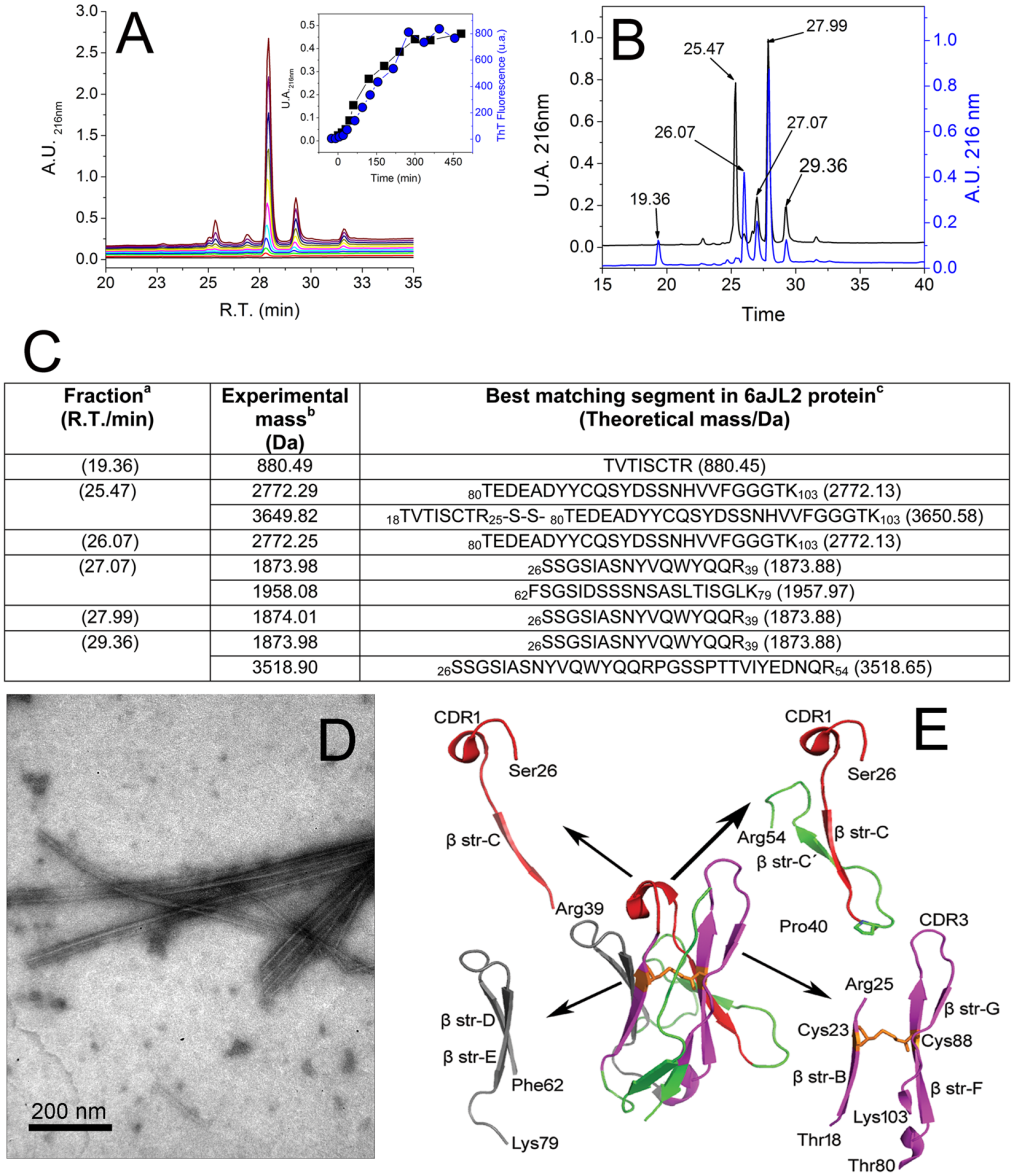
RP-HPLC analysis of (**A**) the aggregates harvested serially during the first seven hours (0, 15 min, 30 min, 45 min, 1 h, 2 h, 3 h, 4 h, 5 h, 6 h and 7 h) and (**B**) after 18 hours of incubation (see Methods) of the products of the proteolysis of 6aJL2 protein with trypsin. The data represented in A and B is the absorbance at 216 nm. In B, the chromatographic profiles of the aggregates analysed in non-reducing (black line) and pre-incubated with 10 mM of DTT (blue line) are shown. Note that the peak with R.T. = 25.47 min, which represent the elution of the fragment Thr18-Arg25-S-S-Thr80-Lys103, is absent in the chromatogram of the reduced sample. Instead, two new peaks appear, with R.T. = 19.36 min and 26.07 min, respectively, which represent the separate elution of the individual peptides. (**C**) Identification of the proteolytic fragments of 6aJL2 protein contained in the fractions of the chromatography separation shown in panels B). ^***a***, ***b***^and ^***c***^mean the same as in Fig. 6. (**D**) Electron micrograph of the aggregates harvested after the overnight incubation of the products of proteolysis of the 6aJL2 protein with trypsin. (**E**) Proteolytic fragments recovered from the aggregates after the overnight incubation of the products of proteolysis of the protein with trypsin, as determined by combining RP-HPLC and MALDI-TOF mass spectrometry. The structural contribution of each fragment to the native 6aJL2 protein is shown with reference to the crystallographic structure of the protein (PDB ID 2W0K).

Once the proteolysis of 6aJL2 protein with trypsin was completed, the products were incubated as described in Methods. The amyloid formation was determined by ThT assay, while the identification of the aggregated fragments was performed by RP-HPLC combined with MALDI-TOF MS (see Methods). The fragment that appeared earliest in the aggregates was Ser26-Arg39, eluted with an average retention time of 27.99 minutes (Fig. 7A). The chromatographic peak corresponding to this fragment showed the largest signal increase (absorbance at 216 nm) during the first seven hours of incubation, matching well with the variation of the ThT fluorescence (Insert at Fig. 7A). This suggests that the aggregation of the fragment Ser26-Arg39 accounted for most of the ThT fluorescence increase observed in that time frame. After 18 hours of incubation, the ThT fluorescence remained constant, suggesting that the fibrillogenesis reaction had reached completion. TEM analysis of the end-point sample showed the presence of fibrillar aggregates (Fig. 7D). RP-HPLC/MALDI-TOF MS analysis showed that the most abundant components of the aggregates were fragments Thr18-Arg25-S-S-Thr80-Lys103, Phe62-Lys79, Ser26-Arg39, and Ser26-Arg54 (Fig. 7B,C). The location of the aggregation-prone fragments into the native structure of 6aJL2 protein is shown in Fig. 7E. Note that fragment Thr18-Arg25-S-S-Thr80-Lys103 is composed by segments Thr18-Arg25 and Thr80-Lys103 linked by the highly conserved intradomain disulphide bond Cys23-Cys88.

### Fibrillogenesis of synthetic peptides with the sequence of the tryptic fragment of 6aJL2 protein

**Figure 8:**
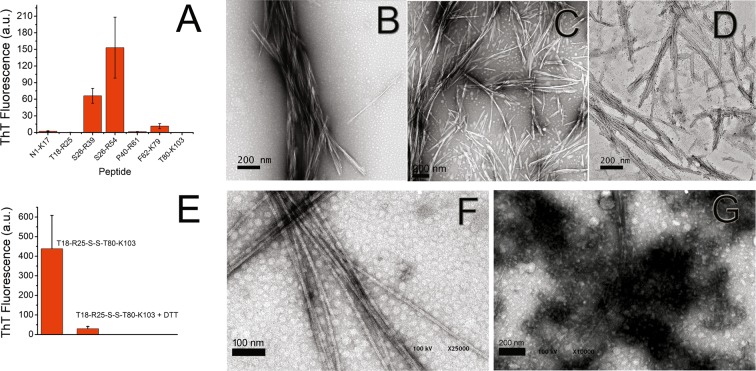
(**A**) Fibrillogenesis assay of the synthetic peptides with the sequence of the tryptic fragments of 6aJL2. Transmission electron micrographs of the aggregates formed by the synthetic peptides (**B**) Ser26-Arg39, (**C**) Ser26-Arg54, and (**D**) Phe62-Lys79 in the aggregation assay shown in panel (A). The images were obtained with a HITACHI-7100 transmission electron microscope. (**E**) Fibrillogenesis assay of the proteolytic fragment Thr18-Arg25-S-S-Thr80-Lys103 incubated in absence (T18-R25-S-S-T80-K103) or in presence of 10 mM DTT (T18-R25-S-S-T80-K103 + DTT). Fragment Thr18-Arg25-S-S-Thr80-Lys103 was generated by proteolysis of soluble 6aJL2 protein with trypsin and then purified by RP-HPLC, as described in Methods. Transmission electron micrographs of the aggregates formed by the tryptic fragment Thr18-Arg25-S-S-Thr80-Lys103 incubated in (**F**) absence or (**G**) in presence of 10 mM DTT, as shown in panel (E). The images were obtained with a CARL ZEISS Libra 120 transmission electron microscope. In (**A**) the data represented is the mean value ± S.D. of thioflavin T fluorescence emission at 482 nm of triplicate samples at the end of the experiment. In (**E**) the data represented is the mean ± 95 C.I. of the thioflavin T fluorescence emission at 482 nm of duplicated samples.

The incubation of the products of proteolysis of 6aJL2 protein with trypsin resulted in the formation of fibrillar aggregates with the properties of amyloid (Fig. 7). This finding indicates that at least one of the tryptic fragments identified in the aggregates can form this type of aggregate. To unambiguously determine which of the tryptic fragments is capable to form amyloid-like fibrils, the aggregation behaviour of seven synthetic peptides with the sequence of the fragments generated by the protease was investigated (Fig. S8). Peptides Ser26-Arg39, Ser26-Arg54 and Phe62-Lys79 formed fibrillar aggregates that give positive ThT fluorescence (Fig. 8A), correlating with the aggregation assay of the tryptic fragments. The far-UV CD spectra of the fibrillar aggregates of peptides Ser26-Arg39 and Phe62-Lys79 is shown in Fig. 4B,D, respectively. Peptides Asn1-Lys17 and Pro40-Arg61 did not form aggregates, which is consistent with the results of the aggregation assay of the tryptic fragments (Fig. 7). Surprisingly, the peptides Thr18-Arg25 and Thr80-Lys103 did not aggregate either (Fig. 8A), an unexpected finding given that they form the disulphide-linked tryptic fragment that was recovered from the aggregates at the end of the incubation (Fig. 7).

### Fibrillogenesis of the tryptic fragment Thr18-Arg25-S-S-Thr80-Lys103 in reducing and non-reducing conditions

The absence of aggregation of the synthetic peptides Thr18-Arg25 and Thr80-Lys103 when incubated separately suggests that the conservation of the disulphide bond Cys23-Cys88 may be a requirement for the aggregation of the whole fragment. To test this hypothesis, the disulphide-linked fragment was purified by RP-HPLC in non-reducing conditions from an overnight digestion of 6aJL2 protein. Then, its aggregation behaviour was evaluated at 150 μM diluted in PBS pH 7.4, in reducing (10 mM of DTT) and non-reducing (absence of DTT) conditions. Fragment Thr18-Arg25-S-S-Thr80-Lys103 incubated in absence of DTT formed long fibrils with ThT binding capacity (Fig. 8E,F), but very scarce fibrils were observed in the sample incubated with DTT (Fig. 8E,G). The far-UV CD spectrum of the fibrils of Thr18-Arg25-S-S-Thr80-Lys103 is characteristic of β-sheet conformation (Fig. 4C). This result shows that, at least in the condition set in this study, the ability of the fragment Thr18-Arg25-S-S-Thr80-Lys103 to form amyloid-like fibrils depends on the conservation of the disulphide bond Cys23-Cys88.

### Scanning Proline (Pro) mutagenesis analysis of Ser26-Arg39 peptide

**Figure 9:**
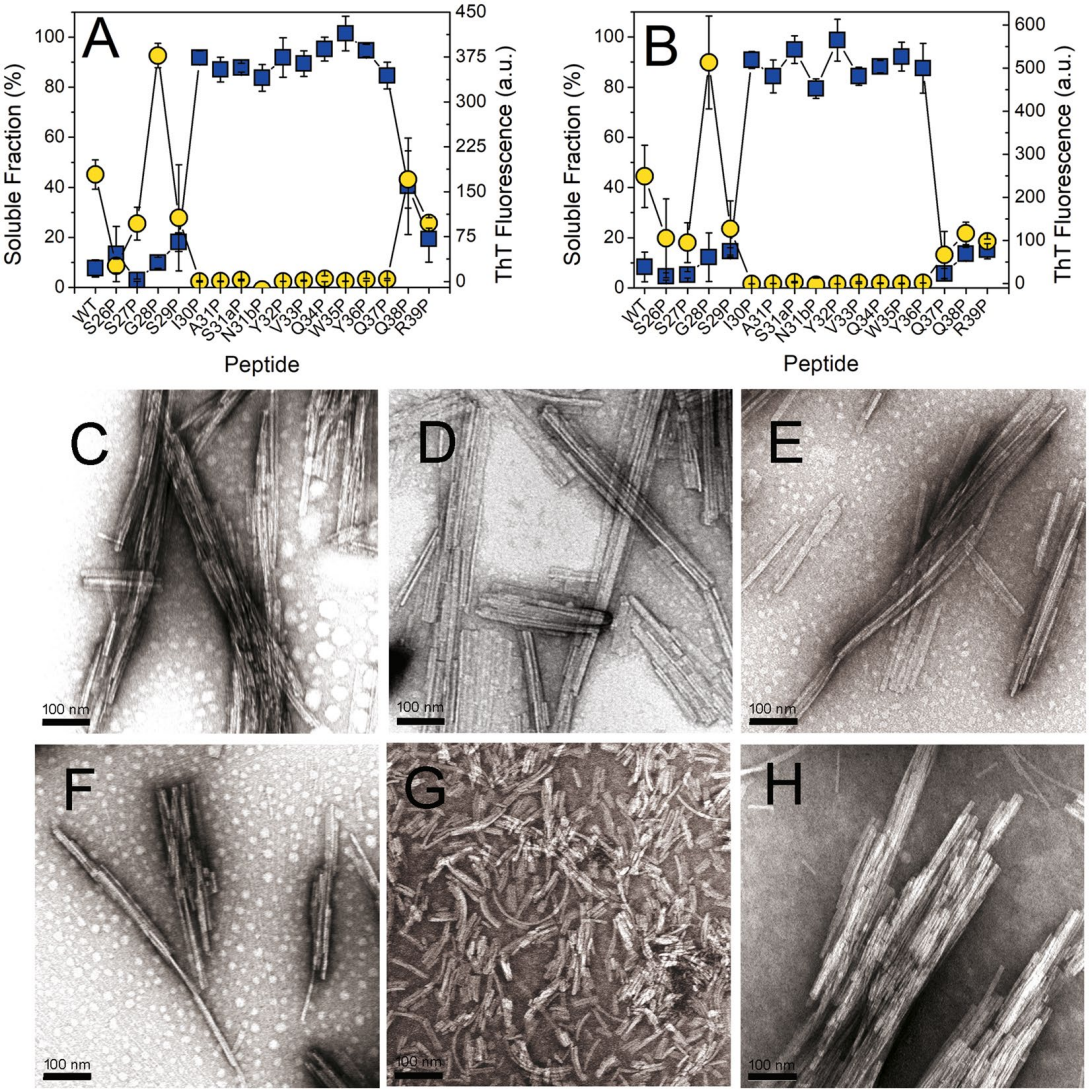
Fibrillogenesis of the single mutants of the highly fibrillogenic peptide Ser26-Arg39 to proline (Scanning proline mutagenesis analysis). Panels (A,B) show the mean value plus the standard deviation of soluble fraction (blue squares) and ThT fluorescence (yellow circles) calculated from triplicates samples after (**A**) 16 hours and (**B**) 40 hours of incubation. Transmission electron micrographs of the aggregates formed by the point mutant peptides (**C**) Ser26Pro, (**D**) Ser27Pro, (**E**) Gly28Pro, (**F**) Ser29Pro, (**G**) Gln38Pro and (**H**) Arg39Pro. The scale bars represent 100 nm. The images were obtained with a HITACHI-7100 transmission electron microscope.

The prediction-based approach was successful in detecting a fibrillogenic sequence spanning from Ile30 to Gln37, which is located in the CDR1 and the β-strand C. This segment is part of the tryptic fragment Ser26-Arg39. Thus, we wanted to identify the residues forming the β-sheet core of Ser26-Arg39 fibrils. For such purpose, a scanning proline mutagenesis analysis was performed with a set of synthetic peptides with single substitutions to Pro (Table S10). Since the wild-type (WT) peptide Ser26-Arg39 showed an extremely high propensity to aggregate upon solubilization in PBS pH 7.4, preliminary aggregation experiments were performed in presence of increasing concentration of GdnHCl for determining the conditions suitable for preparing a stable solution of the peptide (Suppl text S15). It was found that WT peptide Ser26-Arg39 can form amyloid-like fibrils in presence of GdnHCl up to 3.0 M after 40 hours of incubation (Fig. S16). Based on this result, it was decided to perform the aggregation assay with the single mutants to Pro in presence of 2.0 M of GdnHCl. Figure 9A,B show the ThT fluorescence and the Trp fluorescence of the soluble fraction of the samples determined after sixteen and forty hours of incubation, respectively. TEM analysis showed abundant fibrils in all samples that displayed ThT fluorescence. The size and shape of the fibrils were homogeneous in this set (Fig. 9C–F,H), with the notable exception of the mutant Gln38Pro, whose fibrils were shorter and tended to curve, and rather than associate in bundles, they were evenly distributed throughout the grid (Fig. 9G). The data indicate that the region spanning from Ile30 to Tyr36 is sensitive to Pro replacement. Significantly, the replacement of Gln37 to Pro inhibited the aggregation of Ser26-Arg39 after 16 hours of incubation, but not after 40 hours. This suggests that Gln37 is probably at the edge of the Pro-sensitive region.

### *In vitro* fibrillogenesis of the Ser26-Arg39-homologous synthetic peptides

**Figure 10:**
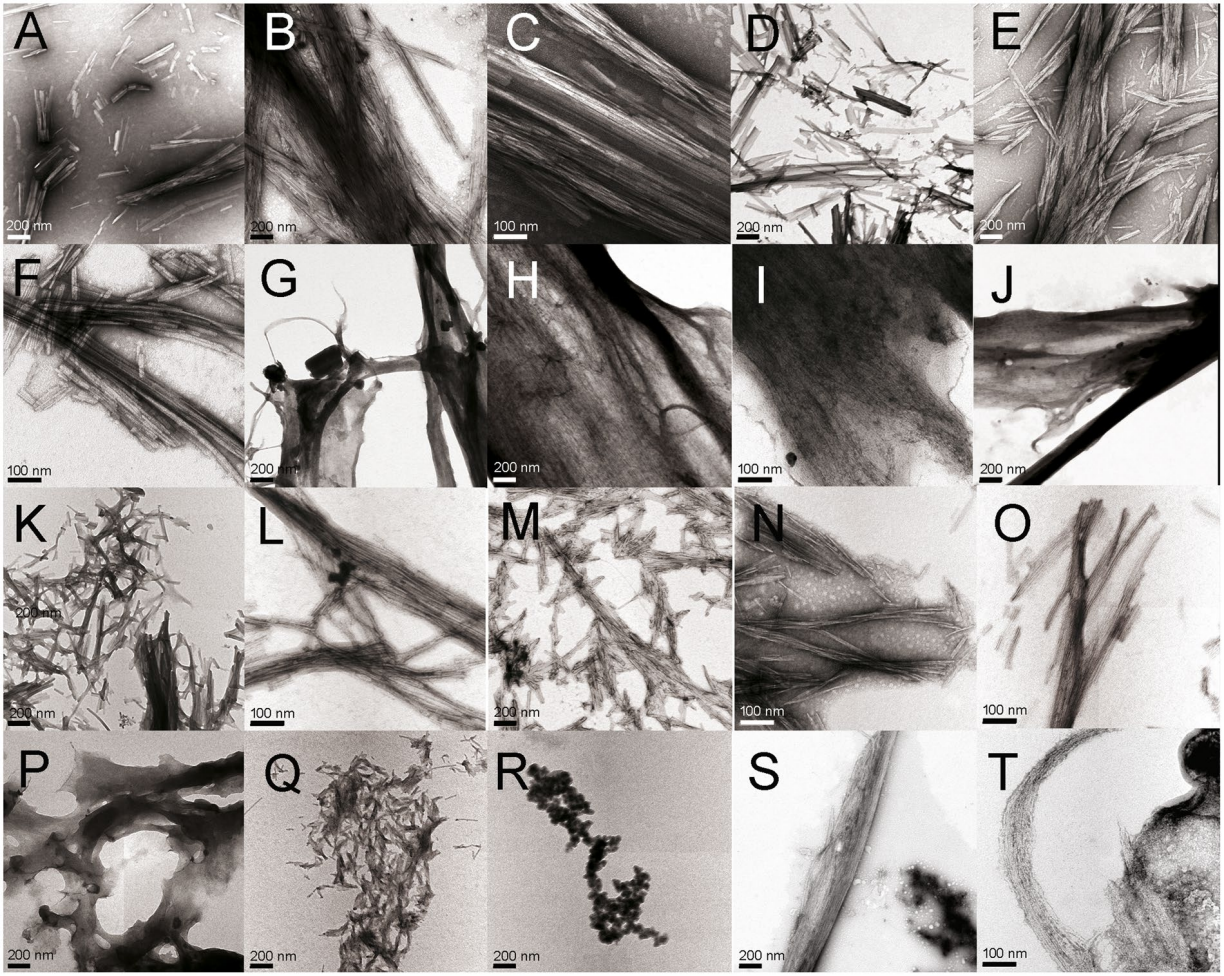
Transmission electron micrographs of the aggregates produced by the synthetic peptides with the sequence from position 26 to 39 of the human light chains, encoded by the V_L_ gene segments (**A**–**C**) *IGLV6-57* (peptide λ6-6a), (**D**–**F**) *IGLV1-36* (peptide λ1-1a), (**G**–**I**) *IGLV1-40* (peptide λ1-1e), (**J**–**L**) *IGLV2-14* (peptide λ2-2a2), (**M**–**O**) *IGKV1-16* (peptide κ1-L1), (**P**–**R**) IGKV1-33 (κ1-018), and (**S**,**T**) *IGKV4-1* (peptide κ4-B3). The peptide sequence is shown in Table S11. The images were obtained with a CARL ZEISS Libra 120 transmission electron microscope.

The finding of a fibrillogenic hotspot in the CDR1 of 6aJL2 protein raised the question whether it is specific to λ6 light chains or is shared by light chains belonging to other V_L_ subgroups. To explore this question, 26 peptides structurally homologous to Ser26-Arg39 were monitored for amyloidogenesis (Table S11). These peptides are encoded by several V_L_ gene segments belonging to the V_L_ subgroups κ1, κ4, λ1, λ2, and λ3, which, together with gene *IGLV6-57*, encode 793 of 808 AL sequences compiled at ALBase (http://albase.bumc.bu.edu/aldb/). The criterium of selection was the frequency of each individual V_L_ gene segments in the AL sequences compiled at ALBase. From each V_L_ subgroups, one gene segment with high frequency, one with middle frequency and one with low frequency, or not present among the AL sequence, were choose. According to the ThT fluorescence assay, five of eleven λ peptides (∼45%) and ten of thirteen κ peptides (∼77%) formed amyloid-like aggregates (Fig. S12). Two third of the peptides belonging to the λ1 and λ2 subgroups formed amyloid. In contrast, only one (3λ−3l) of five λ3 peptides displayed even minor increase in ThT fluorescence. Among the κ peptides, only κ1-A30/L11 and the two κ2 peptides did not form amyloid. Some peptides, as Ser26-Arg39 (identified as λ6-6a), 2-2b2, κ1-L1, κ1-L15/L5/L19 and κ3-L6, appear to aggregate faster than others, since they reached the highest ThT fluorescence value before 24 hours of incubation (Fig. S12). There were also differences between peptides regarding the maximum value of ThT fluorescence reached. In general, good agreement between the variations of ThT fluorescence and the soluble fraction was observed (Fig. S12). TEM analysis established the fibrillar morphology of most aggregates, although extensive structural heterogeneity between samples, as well as intrasample was observed (Fig. 10).

## Discussion

**Figure 11:**
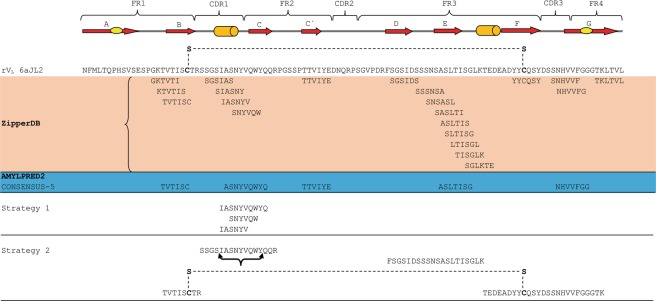
Comparison of the sequences of 6aJL2 protein predicted to be fibrillogenic/aggregation-prone by the computational tools ZipperDB^26^ and AmylPred2^28^ and the fibril-forming segments identified by the two different experimental strategies implemented in this study. Strategy 1 and 2 refer to the “prediction-based” and “proteolysis-based” strategies, respectively, which are described in detail in Methods. The segments of the protein folded as β-strand and α helix are represented as red arrows and green cylinders, respectively. The Framework (FR) and Complementarity Determining Regions (CDR) are indicated at the top of the figure. The sequence of protein 6aJL2 is shown in “one letter” code. The conserved intradomain disulphide bond Cys23-Cys88 is shown as a dashed line in the sequence of 6aJL2 protein and in the tryptic fragment Thr18-Arg25-S-S-Thr80-Lys103. The black curly bracket below the sequence SSGSIASNYVQWYQQR indicates the segment that proved to be sensitive to Pro mutation by scanning proline mutagenesis analysis.

It this study, two complementary approaches were applied to identify the fibrillogenic hot-spots of the rV_L_ 6aJL2 protein. One relied on the predictions of fibrillogenic/aggregation prone sequences performed by two computational tools^26,28^, and the other took advantage of limited proteolysis with trypsin for releasing fragments of 6aJL2 with aggregation-prone sequences, whose aggregation behaviour was subsequently assayed. In general, the computational predictions match well with the experimental results, since most of the sequences predicted to be aggregation prone are within the peptide segments that formed amyloid-like fibrils *in vitro* (Fig. 11). This shows that both tools, ZypperDB and AmylPred2, were able to accurately identify the regions of 6aJL2 protein whose sequence pattern is compatible with a stable amyloid assembly. However, the proteolysis-based strategy was more successful than the prediction-based approach in detecting the aggregation-prone regions. While both strategies identified a fibrillogenic hotspot spanning the CDR1 and β-strand C of the protein, only the proteolysis-based strategy revealed the presence of additional fibrillogenic hotspots in the fragments Thr18-Arg25-S-S-Thr80-Lys103 and Phe62-Lys79. The structural characteristics of these tryptic fragments suggest why the prediction-based strategy failed to detect them. Thr18-Arg25-S-S-Thr80-Lys103 is composed by two peptide segments covalently linked by the disulphide bond Cys23-Cys88 (Fig. 7E). Since the experimental data indicate that the ability of the fragment to form fibrils depends on the conservation of this covalent link (Fig. 8), it can be deduced that the conformational restrictions it imposes contribute critically to the mechanism of fibril assembly and/or stability. It is worth mentioning that some light chains can form amyloid-like aggregates under reducing conditions, which indicates that the preservation of the disulphide bond Cys23-Cys88 is not a general requirement for light chain amyloidogenesis^38,39^. Therefore, in a reducing environment, the fibrillogenesis of at least some light chains appears to be driven by hot spots not dependent on the disulphide bond. One would expect that, under such conditions, the pre-fibrillar and fibrillar aggregates formed would be structurally different from those formed by the molecules with the intact Cys23-Cys88 bond, and most importantly, would exhibit different biological properties.

On the other hand, the dodecamer Phe62-Lys79 comprises the β strands D and E, both of six residues in length (Fig. 7E). As neither of the hexamers predicted to be fibrillogenic within Phe62-Lys79 formed amyloid-like fibrils in our aggregation assay (Fig. 3), it cannot be excluded that the fibrillogenesis of this fragment is driven by cooperative contacts between amyloid stretches located relatively distant in its linear sequence, but that are clustered once aggregation occurs^40^. Fibrillar Phe62-Lys79 may adopt a β arch conformation, a recurring structural motif in both, functional and pathological amyloids^41^, so that the regions corresponding to the β-strands D and E contact each other via their side chains to form a heterosteric zipper and not via the peptide backbone, as occurs in the native protein. Neither of the computational algorithms available for predicting fibrillogenic/aggregation-prone sequences has been optimized for dealing with the complex structural constraints that appear to drive the aggregation of these fragments^34^. For comparative purpose, the peptide concentration (250 μM), incubation time (24 h) and pH (7.4) chosen for assaying the prediction-based peptide library was based on those optimised for testing the aggregation of the tryptic fragments of 6aJL2 protein. It cannot be excluded that such conditions could have limited the capacity of some hexapeptides to aggregate. For example, Ivanova, M.I. *et al*. set peptide concentration and incubation time at 2 mM and up to 45 days respectively, for assaying a set of 59 hexapeptides designed for screening β2-microglobulin and insulin for amyloid-like forming sequences^11^. They found that eight hexapeptides (13.6%) formed amyloid-like aggregates, a higher aggregation rate compared to this study, as only two hexapeptides of 31 formed amyloid-like aggregates (6.5%). However, differences in the incubation buffer (pH = 2.5 in M.I. Ivanova and coworkers^11^), could also contribute to such difference. On the other hand, the relatively stringent conditions used in this study revealed the highly fibrillogenic sequence Ile30-Gln37.

This segment is similar to the sequence stretch found to be sensitive to proline replacement (Fig. 9). It is well known that proline is energetically unfavourable in β-sheet conformations^42,43^, a property that has been successfully used for mapping the secondary structure of the amyloid fibril formed by Aβ(1-40)^44^, and amylin^45^. It can be anticipated that the segment Ile30-Tyr36 is structured in the fibrillar peptide Ser26-Arg39, forming the β-sheet rich fibril core. The high capability of Ile30-Gln37 to self-assemble into fibrils very probably reflects the properties of the hexamer _30b_SNYVQW_35_, as it was shown that this can assemble into highly ordered paracrystalline aggregates, including long cylindrical fibrous crystals (Figs 3F–H, 5D and S5D–F). Such behaviour has been linked to high amyloid propensity^15,26^. The XRFD analysis suggests that, in the crystal lattice, peptide Ser30b-Trp35 arranges as an antiparallel dimer, whose interface is stabilized by a tight homosteric zipper centred on Tyr32 (Fig. 5F). We hypothesize that Ser30b-Trp35 is the driver of the fibrillogenesis of Ser26-Arg39 peptide. Moreover, its strong propensity to form amyloid fibrils could drive larger fragments, and even the entire protein 6aJL2, to adopt destabilized intermediate folding states, favouring amyloid formation. In the native protein, part of Ser26-Arg39 segment (sequence _29_SIASN_31_) folds into a solvent exposed short α-helix, as part of the CDR1, while its C-terminal half extends into the β strand C, buried into the domain (Fig. S13). Such arrangement agrees with what has been observed regarding the spatial positioning of the amyloidogenic determinants in the globular proteins: (1) contrary to that could be expected, they are usually exposed to the solvent^46^, and (2) when exposed at the molecular surface, the amyloid-prone stretches are usually folded into an α-helix, a strategy utilised to hinder the contacts that lead to aggregation^47^. Although part of Ser26-Arg39 folds into an α-helix in the native context, this fragment readily forms β-sheet rich fibrils in isolation, as it was showed by Far-UV CD spectroscopy (Fig. 4B). Such conformational ambivalence agrees with the prediction of similar non-native α-helix or β-sheet propensity that resulted from an analysis performed with CSSP2^48^, a web tool optimized to calculate the contact-dependent secondary structure propensity^49^. It is also supported by the analysis performed with the web application Net-CSSP, optimized to find chameleon sequences in proteins, which detected that two discrete stretches, _29_SIASN_31_ and _30b_SNYVQ_34_, both contained in Ser26-Arg39, are in both α-helical and β-sheet contexts in several native proteins (Fig. S13). This finding strongly suggests that these short sequences are conformationally flexible, being capable to adopt α or β conformation in a context-dependent manner, a hallmark of the chameleon sequences^50^. Hence, both theoretical and experimental data suggest that Ser26-Arg39 is a conformationally flexible segment, containing sequence element that could be the “switch” triggering the α-helix to β-strand transition of the CDR1. Such transition could be part of a more general structural adjustment that could drive the self-assembly of 6aJL2 protein into amyloid-like fibrils. It is believed that α-helix to β-strand transitions driven by chameleon sequences play a key role in the amyloidogenesis of α helical proteins, as insulin^46^, apomyoglobulin^51^, and ApoA-I^52^.

The data obtained in this study prove the presence of a strongly fibrillogenic hot-spot spanning the CDR1 and β-strand C of 6aJL2. The fibrillar state promoted by such hot-spot is highly stable, since peptide Ser26-Arg39 was able to form fibrils even in 3.0 M of GdnHCl (Fig. S16). Consider that the C_*m*_ of reversible unfolding of 6aJL2 protein in GdnHCl at 25 °C is 1.4 M^17^. This finding is consistent with the high propensity of the 6aJL2 protein to aggregate as amyloid-like fibrils *in vitro*. It has been shown that this protein efficiently forms fibrils under near physiological conditions of temperature, pH and ionic strength^17,53^. In addition, it was recently demonstrated that 6aJL2 and its point mutant 6aJL2-R25G are the proteins most prone to aggregation among a group of six rV_L_ proteins encoded by the germ line of five V_L_ gene segments that are associated to amyloid deposition^24^. It should be noted that 6aJL2 has the germline sequence of subgroup λ6, the family of light chains that shows the strongest association with amyloid deposition^18–21,24^. Hence, sequence elements contained in the segment Ile30-Gln37 are obvious candidates for the driver of the amyloid aggregation of 6aJL2. However, the role played by the aggregation-prone stretches in globular proteins depend not only on their “individual intrinsic aggregability”, but also accessibility to the molecular surface^46,47^, restriction imposed by the 3D folding of the protein^54^, and, in the case of the light chain, the effect of the somatic mutations^55^, could be dominants. Thus, it cannot be excluded that the hot-spot revealed in the fragments Thr18-Arg25-S-S-Thr80-Lys103 and Phe62-Lys79 play an important role in the fibrillogenesis of 6aJL2 protein, as well as in general for the λ6 light chains, representing potential target for theranostics agents.

In this study, the aggregation of 24 synthetic peptides structurally homologous to Ser26-Arg39, encoded by 34 different V_L_ gene segments, 23 κ and 11 λ, was monitored (Table S11). They represent roughly 50% of the sequence diversity encoded by the human germline V_L_ repertoire^56^. It was found that 15 of them (60%) form fibrillar aggregates that gave positive ThT fluorescence (Fig. S12). This finding shows that a fibrillogenic hotspot associated to the CDR1 is a feature shared by light chains belonging to different κ and λ subgroups. Moreover, since the structure of the CDR1 is determined by both germline-encoded sequence elements as well as variations introduced by somatic hypermutation^57,58^, this finding also suggests a link between the evolution of the light chain gene segments and the mechanisms of diversification of antibody repertoire, on one hand, and the marked difference in amyloid propensity and pathogenic variability that characterizes light chains^18,19,59,60^, on the other. In this regard, comparative sequence alignment suggests that the inability of some Ser26-Arg39-homologous peptides to form fibrils is better explained by the presence of one or more charged residues (Asp, Glu, Arg and Lys) at positions potentially critical for fibril assembly (Fig. S14). The placement of such residues at a position involved in the fibril core would impose a desolvation energy penalty for self-assembly into fibrils that for some peptides could be large enough as to disfavour aggregation^37,54^. TEM analysis revealed a wide morphological heterogeneity of the aggregates formed by these peptides (Fig. 10), a finding that can be expected, given their large structural heterogeneity, both in length and in sequence (Fig. S14). Interestingly, it appears not to be a correlation between the propensity of the peptides to aggregate as amyloid-like fibrils and the frequency of the V_L_ gene segment encoding them in AL amyloidosis (Table S11). In fact, it was found that the fraction of fibril-forming peptides of κ type (77%) was higher than that of λ type (41.6%), which contrasts with the well-known higher frequency of λ light chains in amyloidosis^61^. Moreover, peptide λ3-3r, encoded by the AL-associated gene segment 3r (*IGLV3-1*)^20^, did not form fibrils. These findings, in conjunction with others of this study, support the concept that the amyloidogenesis of the light chains is a complex process in which more than one amyloid-prone hotspot could drive the aggregation. Such concept is in line with the results of recent studies that used solid state nuclear magnetic resonance analysis for characterizing light chain fibrils, since they found that the segments of the protein that form the fibril core vary from one light chain to another^62–64^. Differences in the fibril core arrangement should be expected to translate into heterogeneity in morphology and biological properties of the aggregates. The interplay between the structural heterogeneity of the light chains and the amyloid aggregates that they form, on the one hand, and the genetic background of the patients on the other, would explain, at least in part, the great clinical heterogeneity that characterizes AL amyloidosis^59^. In agreement with this, it has been found that the usage of the repertoire of V_L_ gene segments is linked to key clinical aspect of the AL amyloidosis^18,19,65^. Finally, this study could open the door to novel therapeutic approaches of AL amyloidosis, since the aggregation hotspots identified represent potential targets of ligands that could inhibit the amyloid aggregation of the light chains.

## Conclusions

1. Prediction-based approaches like those used in this study, could fail to detect fibrillogenic hotspots in protein’s regions that assemble into fibrils by mechanisms more complex than that driving the aggregation of self-complementary short peptides.
2. The λ6 rV_L_ protein 6aJL2 has several fibrillogenic hotspots. The hotspot spanning the CDR1 and the β-strand C is the most fibrillogenic of all.
3. A fibrillogenic hotspot associated to the CDR1 is also present in other light chains belonging to different κ and λ V_L_ subgroup.

## Methods

### Protein cloning, expression and purification

Cloning, bacterial expression and chromatographic purification of the germline λ6 rV_L_ 6aJL2 protein has been described elsewhere^17^.

### Identification of the fibrillogenic regions

Two different approaches were used to identify fibrillogenic/aggregation-prone sequences in 6aJL2 protein (Fig. S1). In one of them, from now on named “*prediction-based strategy”*, the sequence of 6aJL2 protein was screened with two different web-based computational tools, Zipper-DB (http://services.mbi.ucla.edu/zipperdb/), and AmylPred-2 (http://aias.biol.uoa.gr/AMYLPRED2/). The first one computes fibrillogenic propensity using the structural-based algorithm 3D-profile method^26^. The second one is a consensus method for predicting aggregation-prone regions in globular protein^28^. Then, the predictions generated by both tools were tested with a set of synthetic peptides named “*prediction-based peptide library”*, composed by 31 hexapeptides and one decapeptide (Table S2 and Fig. 3)^29^. In the second approach, known as “*proteolysis-based strategy*”, 6aJL2 protein was proteolyzed with trypsin, and the products incubated to promote the aggregation. The aggregates were collected by centrifugation at 14,000 r.p.m. in a table-top centrifuge, resolubilized in 6.0 M guanidine hydrochloride (GdnHCl) and the components identified by combining RP-HPLC and MALDI-TOF mass spectrometry. Finally, the results were validated with a set of synthetic peptides with the sequence of the tryptic fragments (Fig. S8).

### Aggregation assay with the prediction-based peptide library

Aliquots (300 µl) of synthetic peptides dilutions (250 µM) in phosphate-buffered saline (PBS) pH 7.4 plus 0.05% w/v sodium azide were deposited in 2 mL capped polypropylene microcentrifuge tubes (AXYGEN, Corning, NY, USA, Cat. No. MCT-200-C) and incubated for 24 hours at 37 °C with constant orbital shaking of 1000 r.p.m in a Thermomixer Comfort (EPPENDORF AG, Hamburg, Germany)^29^. The presence of amyloid-like aggregates was determined by the thioflavin T (ThT) fluorescence assay^66^. An aliquot of the endpoint samples was withdrawn and stored at 4 °C for ultrastructural analysis by transmission electron microscopy (TEM). The same conditions were used to test the aggregation of the set of peptides with the sequence of the tryptic fragments of 6aJL2 (Fig. S8). The synthetic peptides used in this study were purchased to GenScript (Genscript Biotech Corp., Piscataway, NJ 08854, USA) and they are more than 85% pure. Both purity and the correct mass of each peptide were determined by the provider by RP-HPLC and mass spectrometry analysis, respectively, as part of the normal quality control. A complete report of the process of quality evaluation of each peptide was provided to us by the GenScript.

### Proteolysis of 6aJL2 with trypsin and fibrillogenesis of the tryptic fragments

A 3 mg/ml (250 µM) protein dilution in Tris-HCl 50 mM pH 8.0 was added with Trypsin Gold, mass spectrometry grade (Promega Corporation, Madison, USA) at a w/w protein/trypsin ratio of 200/1. Samples were incubated overnight (~16 h) at 37 °C without agitation and then centrifuged at 25,000 r.p.m. at 4 °C for 30 min to sediment all preformed aggregates. An aliquot (600 µl) of the supernatant was carefully recovered and deposited in a 2 mL capped polypropylene microcentrifuge tubes (AXYGEN, Corning, NY, USA, Cat. No. MCT-200-C) and the aggregation assay was performed as described for the prediction-based peptide library, except that the agitation speed was set at 500 r.p.m. At time zero and at different incubation times, an aliquot (20 µl) was taken and the presence of amyloid aggregates determined by the ThT fluorescence assay^66^. At the same time, a second aliquot (50 µl) was withdrawn from the sample and centrifuged 25,000 g at 4 °C for 30 min to sediment the aggregates. The supernatant was carefully withdrawn, the pelleted aggregates washed with 100 µl of PBS pH 7.4, and sedimented again by centrifugation. The supernatant was discarded, and the pelleted aggregates resuspended in 100 µl of 6 M GdnHCl solution in miliQ water, with an incubation step of 15–20 min at 75 °C. Then, the sample was centrifuged as described before to eliminate any persisting aggregate and the supernatant was injected into an analytical Vydac 218TP53 C18 reverse phase column (GRACE, Columbia, Maryland, USA), installed in a Waters Alliance 2695 HPLC system (Waters Corporation, Milford, Maryland, USA). The components absorbed to the column were eluted with a gradient of 0–60% of acetonitrile with 0.1% (v/v) TFA (solvent B) in miliQ-grade water with 0.1% (v/v) TFA (solvent A) in 60 min, keeping the flow at 0.5 ml/min. The eluting fractions were detected by absorbance at 216 nm and fluorescence emission (excitation and emission wavelength of 295 nm and 350 nm, respectively) and were collected manually, dried in a rotary evaporator, and resuspended in an adequate volume. Then, they were mixed with the appropriated matrix, and the mass of the components of each fraction were determined by mass spectrometry in a 4800 PLUS MALDI TOF/TOF Analyzer (Applied Biosystems Inc, Foster City, California, USA).

### Purification and fibrillogenesis of the recombinant fragment Thr18-Arg25-Thr80-lys103

Proteolysis of 6aJL2 protein with trypsin, fibrillogenesis of the product of proteolysis, and purification of the recombinant fragment Thr18-Arg25-Thr80-Lys103 from the aggregates were performed as described in the previous paragraph. Once purified by RP-HPLC chromatography, the aggregation assay of the recombinant tryptic fragment was performed as described for the prediction-based peptide library, with two exceptions: (1) the concentration of the peptide was set at 150 μM; and (2) the aggregation of the fragment was evaluated in non-reducing (absence of DTT) and reducing (presence of 10 mM of DTT) conditions. The samples were assayed in duplicate.

### Preparation of stable stock solution of the synthetic peptide Ser26-Arg39

All attempts to prepare a stable solution of the synthetic peptide Ser26-Arg39 in PBS pH 7.5 or miliQ water were unsuccessful, since the peptide aggregates almost instantly. To solve this problem, the effect of GdnHCl on the solubility and aggregation of the peptide Ser26-Arg39 was investigated (Supplementary text S15). It was found that the peptide Ser26-Arg39 forms amyloid-like aggregates in the presence of GdnHCl up to 3.0 M, being the aggregation totally inhibited at higher concentration of the denaturant (Fig. S16). Hence, the stock solution of the peptide Ser26-Arg39, as well as that of any other peptide whose aggregation would be compared to it, were prepared in PBS pH 7.4 plus 6.0 M GdnHCl. The peptide concentration was determined by UV spectroscopy, using the molar extinction coefficient at 280 nm calculated from the peptide sequence by the web-based tool PROTPARAM (https://web.expasy.org/protparam/). Then, working samples were prepared by diluting the stock solutions to reach the required GdnHCl concentration.

### Scanning proline mutagenesis of the wild-type peptide Ser26-Arg39

The fibrillogenesis was monitored for the single mutants of peptide Ser26-Arg39 to Pro (Table S5) as described for the prediction-based peptide library, with the exception that the peptides were diluted at 150 µM in PBS pH 7.4 plus GdnHCl 2 M and the speed of orbital shaking was set at 500 r.p.m. Each peptide was assayed in triplicate. After 16 h and 40 h of incubation, an aliquot (50 µl) was taken from each sample and the presence of amyloid-like aggregates was determined by the ThT fluorometric assay^66^. In parallel, a second aliquot was removed from the sample and centrifuged at 25,000 g for 30 min at 4 °C. An aliquot (20 µl) of the supernatant was carefully withdrawn, avoiding contamination of the sedimented aggregates, and mixed with 1 ml of PBS pH 7.4. The intrinsic fluorescence of the sample was registered in the range from 310 nm to 410 nm, exciting the sample at 280 nm. The excitation and emission slits were set at 2 nm. Integration was performed every 1 nm. The aggregated fraction (*A*.*F*.*)* of each peptide at the time of analysis was calculated by the equation 1:

1 $$A.F.=1-({\rm{I}}.{\rm{F}}{.}^{t}/{\rm{I}}.{\rm{F}}{.}^{t{0}})$$

where I.F.^*t*^ and I.F.^*t0*^ are the intrinsic fluorescence at a particular incubation time and time zero, respectively.

### Aggregation assay of the set of Ser26-Arg39-homologous synthetic peptides

The amyloid-forming propensity of twenty-five synthetic peptides structurally homologous to peptide Ser26-Arg39, which are encoded by twelve λ and twenty-six κ germline V_L_ gene segments was evaluated (Table S11). The fibrillogenesis assay was performed as described for the prediction-based peptide library, with the exception that the peptide concentration was 150 µM, diluted in PBS pH 7.4 plus GdnHCl 1 M, and the speed of orbital shaking was set at 500 r.p.m. Each peptide was assayed in duplicate. After 24 hours of incubation, the presence of amyloid-like aggregates was determined by the ThT fluorescence assay^66^.

### ThT fluorescence assay

The aliquots taken from the samples were mixed with 2 ml of a 20 µM ThT dilution in 50 mM TrisHCl pH 8.0. The ThT fluorescence, indicative of the presence of amyloid aggregates, was determined in the interval from 460 nm to 510 nm, exciting the sample at 450 nm. The excitation and emission slits were set at 2 nm. Integration was performed every 1 nm. All measurements of fluorescence (ThT and intrinsic fluorescence) were performed in a FluoroLog-3 spectrofluorometer (HORIBA INSTRUMENTS INC., Albany, New York, USA).

### Transmission Electron Microscopy (TEM)

The sample grids were prepared as previously described^17^. TEM images were obtained using a CARL ZEISS Libra 120 (Carl Zeiss AG, Oberkochen, Germany) or a HITACHI-7100 (Hitachi, Ltd, Tokyo, Japan) transmission electron microscope, in both cases running at 100 kV, coupled with a Gatan Ultrascan 1000 CCD (2000 × 2000 pixels) camera (GATAN, Pleasanton, California, USA) to record the images.

### X-Ray Fiber Diffraction (XRFD)

To produce material suitable for XRFD analysis, the peptides Ile30-Val33, Ser30b-Trp35, and Ile30-Gln37 were aggregated under the same conditions described for the prediction-based peptide library, but, instead of in PBS pH 7.4, the peptides were solubilized in milli-Q water plus 0.05% Na azide. The aggregates showing fibrillar characteristics after TEM imaging were aligned to obtain a fibrous-textured fibril prior to XRFD, according to method described previously^67^. XRFD was carried out in-house using a RIGAKU 007 F CuKα rotating anode X-ray source (Rigaku Corporation, Tokio, Japan) running at 40 kW and 30 mA and a Saturn 944 + CCD detector (Rigaku Corporation, Tokio, Japan) (λ = 1.5419 Å, sample to detector distance 50 and100 mm). The sample was placed upright in the path of the X-ray beam on a goniometer head, matching the fibre axis with the rotation axis of the sample plate. Diffraction data were initially observed using Mosflm^68^ and images were output as TIF files for further analysis. CLEARER software was used to measure the location of the diffraction peaks and to explore the dimensions of the unit cell. Raw diffraction patterns were centred and radially averaged prior to peak searching with the following settings: sample to detector plate distance D = 100 mm, default wavelength λ = 1.5419 Å and a pixel size of 179.1 µm. All other settings were set as default. Only the most intense peaks found using CLEARER were used for the unit cell prediction, and the initial guess for the unit cell was set as *a* = 4.7 Å *b* = 20 Å *c* = 20 Å, with *a* set as the fibre axis, *b* as the inter-sheet spacing and *c* as the chain length. The unit cell dimensions were optimized for *b* and *c*. For the diffraction pattern prediction, the same settings were used, as well as a fibre disorder angle of 0.4 radians and a sampling interval of 1 pixel. The fibre axis was set parallel to the meridional axis of the diffraction patterns and the beam axis was set to be perpendicular to that axis. The calculated diffraction pattern generated from the determined unit cell was then compared back to the observed pattern, by superimposing a quadrant of the predicted pattern over the experimental diffraction pattern.

### Far-UV Circular dichroism (CD) spectroscopy

Far-V CD was performed at 25 °C in a JASCO 710 Spectropolarimeter (JASCO, Easton, MD 21601, USA) using a quartz cell with a light path of 0.1 cm, as described previously^17^.

## Supplementary information

Supplementary information
