# Supplementary material for: The CDR1 and Other Regions of Immunoglobulin Light Chains are Hot Spots for Amyloid Aggregation
Source: Sci Rep. 2019 Feb 28;9:3123. doi: 10.1038/s41598-019-39781-3 (PMC6395779; doi:10.1038/s41598-019-39781-3)
Supplement: Supplementary file 1 — Supplementary information [file 41598_2019_39781_MOESM1_ESM.pdf]

SUPPLEMENTARY INFORMATION

The CDR1 And Other Regions Of Immunoglobulin Light Chains Are Hot Spots For Amyloid Aggregation

Robin Axel Ruiz-Zamora[1], Simon Guillaumé[2], Youssra K. Al-Hilaly[2,3], Zahraa Al-Garawi[2,3], Francisco Javier Rodríguez-Alvarez[1], Guadalupe Zavala-Padilla[4], Julio I. Pérez-Carreón[1], Sandra L. Rodríguez-Ambriz[5], Guillermo A. Herrera[6], Baltazar Becerril-Luján[4], Adrián Ochoa-Leyva[4], Jorge Melendez-Zajgla[1], Louise Serpell[2], Luis del Pozo-Yauner[1,6*].

[1] Instituto Nacional de Medicina Genómica, Ciudad de México, México.
[2] School of Life Sciences, University of Sussex, Falmer, Brighton, East Sussex, BN1 9QG, United Kingdom.

[3] Chemistry Department, College of Science, Mustansiriyah University, Baghdad, Iraq.

[4] Instituto de Biotecnología Universidad Nacional Autónoma de México (UNAM), Cuernavaca, Morelos, México.

[5] Centro de Desarrollo de Productos Bióticos, Instituto Politécnico Nacional, Yautepec, Morelos, México.

[6] Department of Pathology and Translational Pathobiology, LSU Health Sciences Center Shreveport, 1501 Kings Hwy, Shreveport, LA 71103.

* Corresponding author

Instituto Nacional de Medicina Genómica, Ciudad de México, México.

Present address:

Department of Pathology and Translational Pathobiology, Louisiana State University Health Sciences Center, Shreveport, LA 71103, USA

Phone: (318) 626 1218

Email: ldelpo@lsuhsc.edu

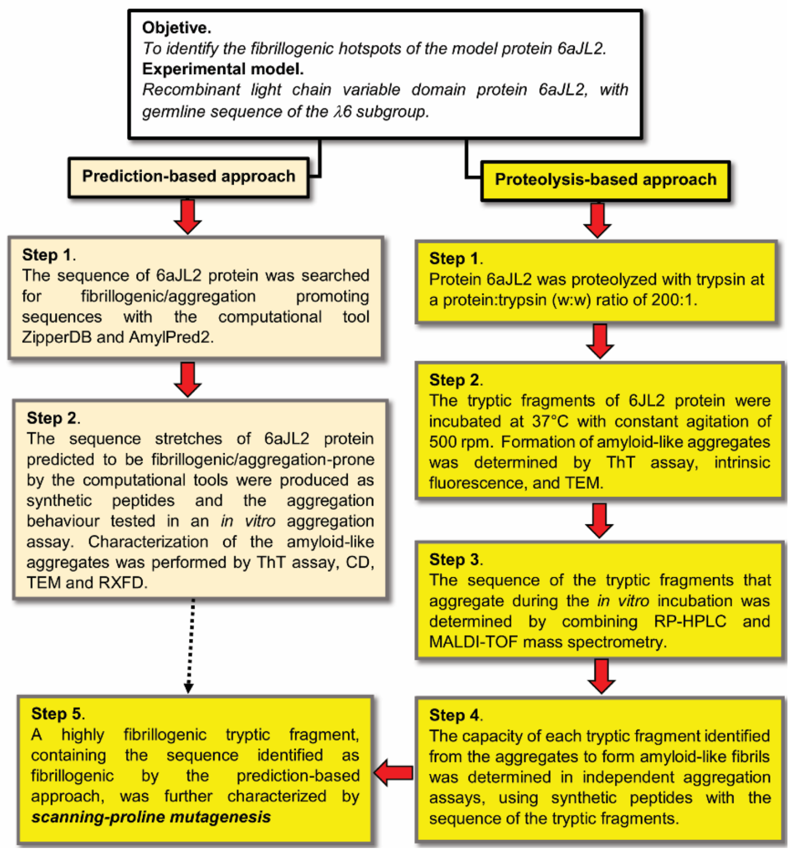

Figure S1. Experimental strategy applied to identified profibrillogenic hot-spots in the rV$_L$ protein 6aJL2. Two complementary approaches were used. One was based on computational algorithms optimized to predict fibrillogenic/aggregation-prone sequences based on different structural and biophysical properties of the polypeptide chain. The second approach was based on limited proteolysis of 6aJL2 protein and the subsequent identification of the aggregation competent fragments. **Legend**: ThT: thioflavin T, CD: circular dichroism, TEM: transmission electron microscopy, and XRFD: X ray fibril diffraction.

Table S2. Library of thirty-two synthetic peptides (thirty-one hexapeptides and one decapeptide) designed for testing the predictions of the web-based computational tools ZipperDB and AmylPred2[1]. The library is composed by all the hexamers with Rosetta energy of -23 kcal/mol or lower[2] (21 sequence, of which 8 have a Rosetta energy of -25.5 kcal/mol or lower[3]), which are predicted to be prone to form fibrils (highlighted in yellow), and 10 hexamers with Rosetta energy higher than -23.0 kcal/mol, predicted by ZipperDB to have low propensity to form amyloid. The group of 10 hexamers were not selected randomly but were selected for their sequence similarity with the hexamers identified as potentially fibrillogenic, differing from them in one or two positions by the displacement of the search window[2].

No.	Peptide I.D.	Sequence (one letter code)	Rosetta energy[1] (kcal/mol)	No.	Peptide I.D.	Sequence (one letter code)	Rosetta energy[1] (kcal/mol)
1	L6(N1-Q6)	NFMLTQ	-22.3	17	L6(S70-I75)	SASLTI	-24.9
2	L6(H8-S14)	HSVSES	-22.5	18	L6(A71-S76)	ASLTIS	-25.2
3	L6(G16-I21)	GKTVTI	-27.5	19	L6(S72-G77)	SLTISG	-26.0
4	L6(K17-S22)	KTVTIS	-26.0	20	L6(L73-L78)	LTISGL	-24.3
5	L6(T18-C23)	TVTISC	-27.3	21	L6(T74-K79)	TISGLK	-23.2
6	L6(T20-R25)	TISCTR	-21.4	22	L6(I75-T80)	ISGLKT	-18.8
7	L6(S27-S31a)	SGSIAS	-25.9	23	L6(S76-E81)	SGLKTE	-24.2
8	L6(S29-Y32)	SIASNY	-26.3	24	L6(T80-D85)	TEDEAD	-16.2
9	L6(I30-V33)	IASNYV	-23.5	25	L6(E81-Y86)	EDEADY	-14.5
10	L6(S30b-W35)	SNYVQW	-24.9	26	L6(Y86-Y91)	YYCQSY	-26.3
11	L6(Y32-Q37)	YVQWYQ	-20.3	27	L6(Y87-D92)	YCQSYD	-21.5
12	L6(T45-E50)	TTVIYE	-23.1	28	L6(S94-F98)	SNHVVF	-25.9
13	L6(S63-S68)	SGSIDS	-24.7	29	L6(N95-G99)	NHVVFG	-24.1
14	L6(S68-A71)	SSSNSA	-24.4	30	L6(H95a-G100)	HVVFGG	-20.0
15	L6(S68b-L73)	SNSASL	-25.3	31	L6(T102-L017)	TKLTVL	-25.1
16	L6(N69-T74)	NSASLT	-21.8	32	L6(I30-Q37)	IASNYVQWYQ	-

[1] Calculated using the RosettaDesign program[4]. Segments with energies equal to or below -23 kcal/mol are deemed to have high fibrillation propensity[2].

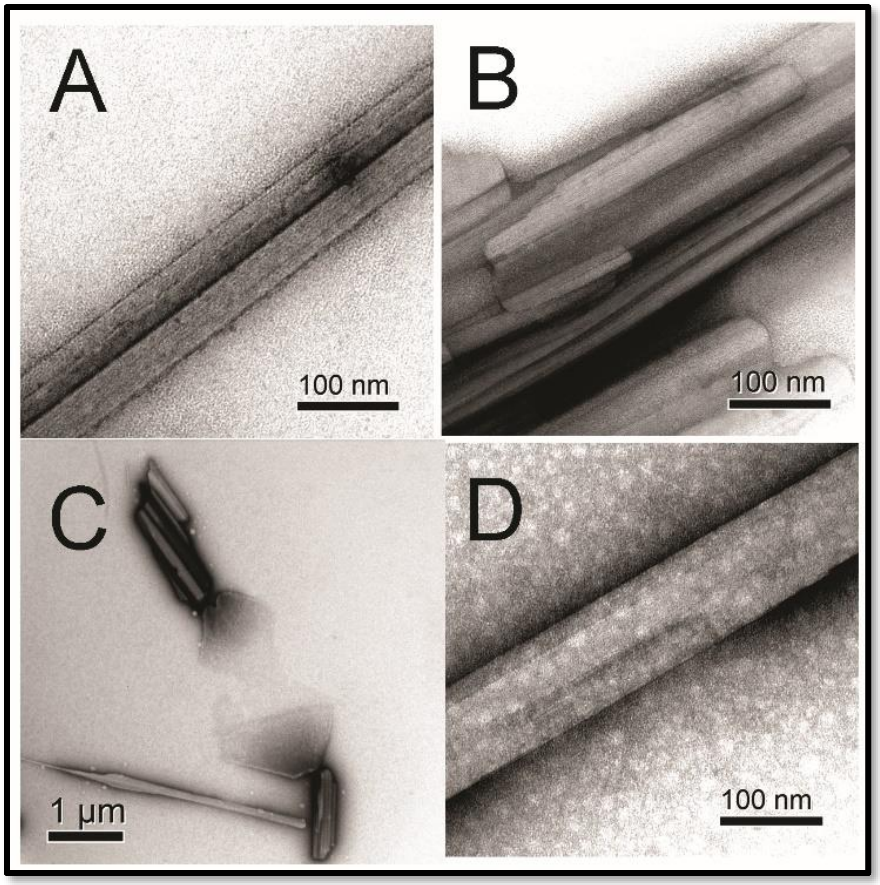

Figure S3. Transmission electron micrographs of the aggregates formed by the synthetic peptides Ile30-Gln37 (**A** & **B**) and Ser30b-Trp35 (**C** & **D**), diluted at 250 µM in PBS pH 7.4 plus 0.05% Na azide, and incubated at 37°C with constant agitation for 24 hours.

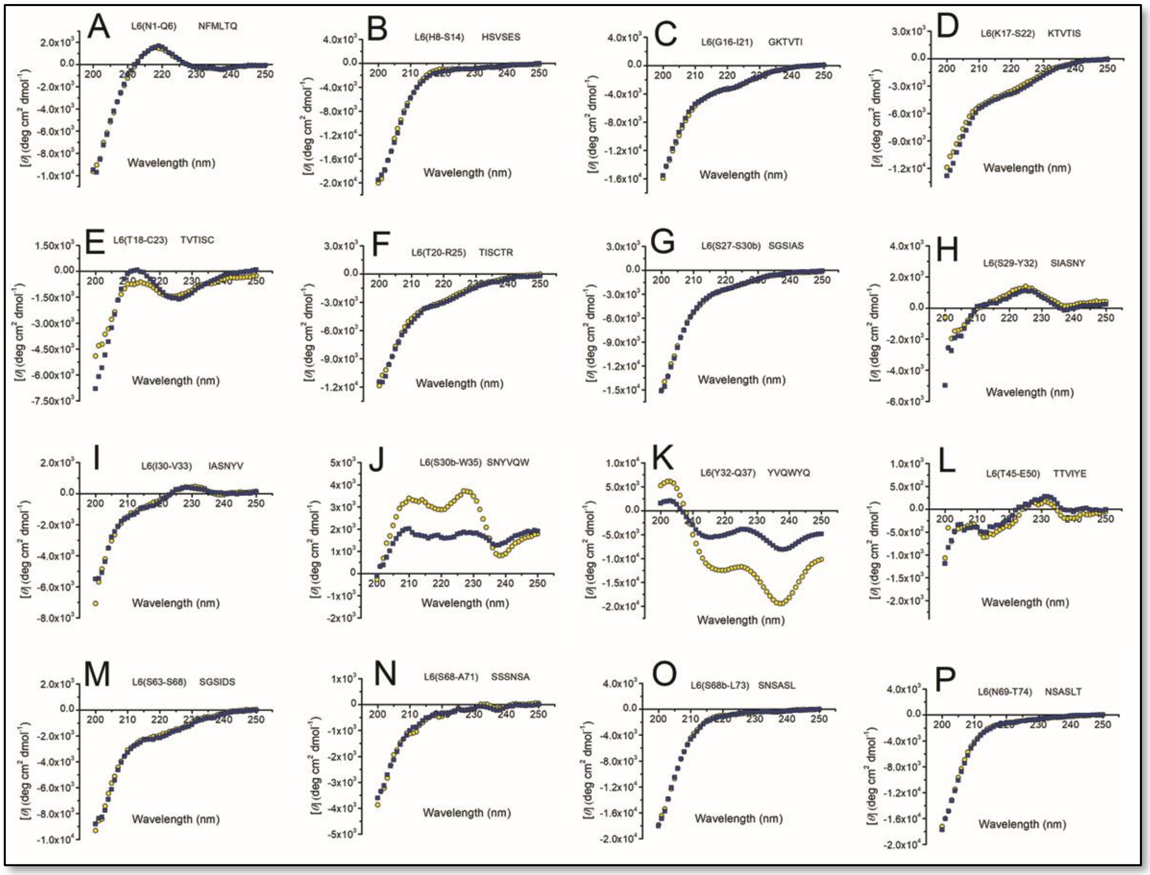

Figure S4. It continues the next page.

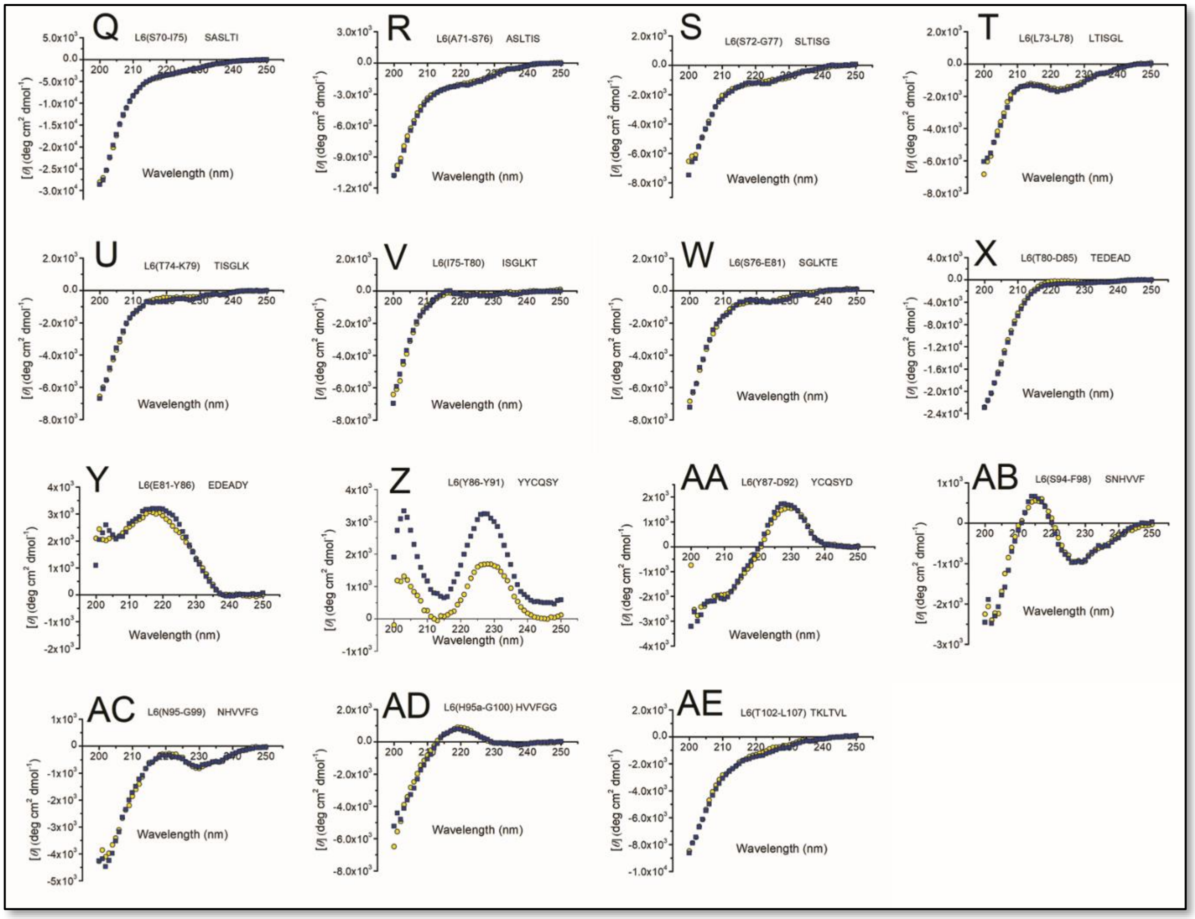

Figure S4. Far-UV circular dichroism spectra of hexapeptides (250 µM) diluted in PBS pH 7.4 before (yellow circle) and after (blue square) 22 hours of incubation at 37°C with constant orbital shaking at 1000 r.p.m. Far-UV CD spectroscopy was performed at 25°C in a Jasco 710 Spectropolarimeter (JASCO Easton, MD) using a quartz cell with a light path of 0.1 cm. The spectra were registered in the range of 190–260 nm, at a scan rate of 20 nm/min, and a band width of 1 nm. The parameters data pitch and response time were set at 1 nm and 8 sec., respectively. For each sample, two consecutive spectra were obtained, averaged, and baseline-subtracted.

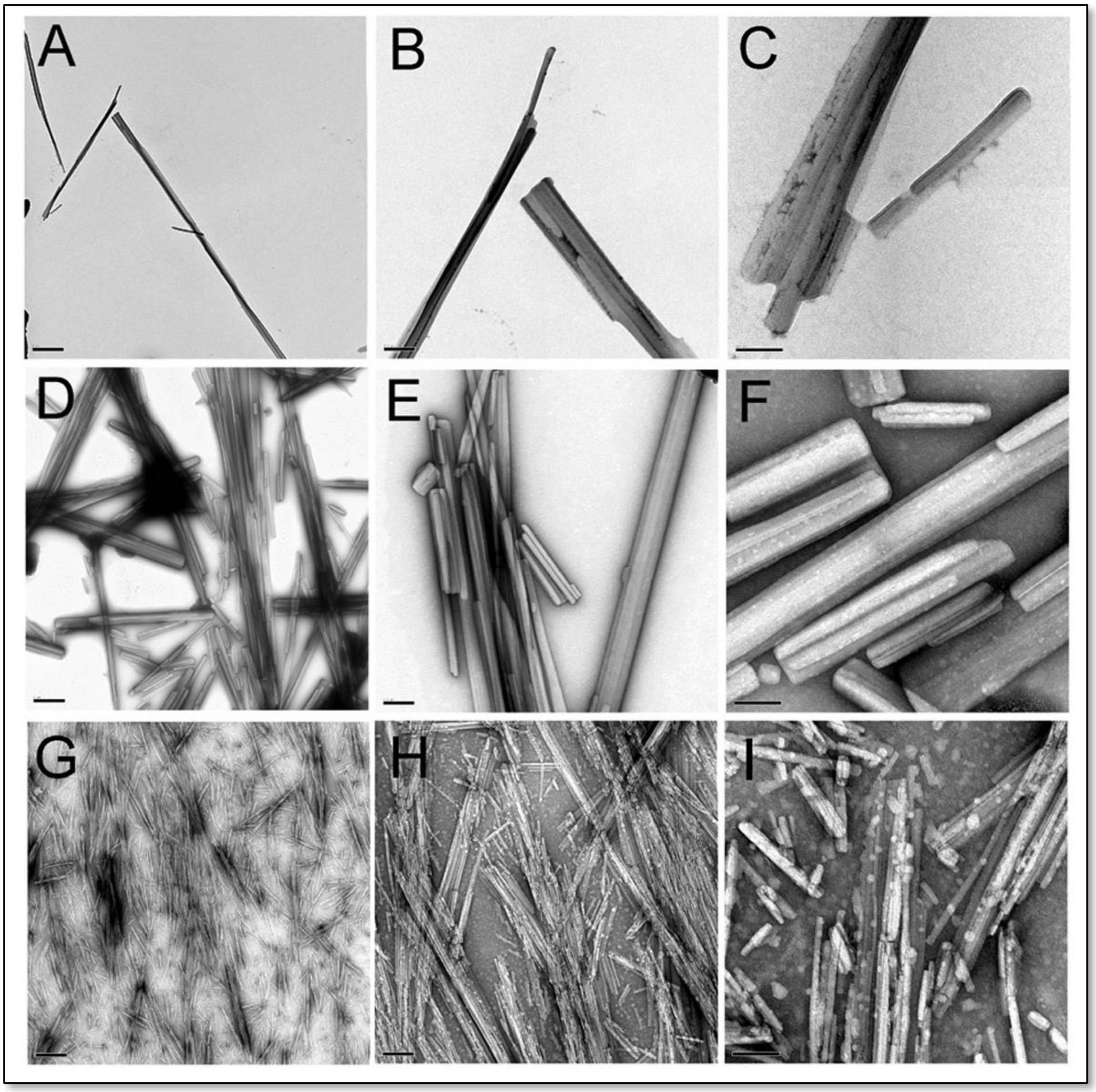

Figure S5. Transmission electron micrographs of the aggregates formed by the synthetic peptides Ile30-Val33 **(A-C)**, Ser30b-Trp35 **(D-F)** and Ile30-Gln37 **(G-I)** diluted 250 µM in miliQ water plus 0.05% Na Azide and incubated at 37°C with constant agitation. The scale bars in panels A, D, and G represent 1µm, in panels B, E, and H represent 0.2 µm and in panels C, F, and I represent 0.1 µm.

Table S6. Position of the diffraction signals observed in the XRFD patterns generated by the fibrillar aggregates of peptides Ser30b-Trp35 and Ile30-Gln37.

Ser30b-Trp35	Ile30-Gln37
**Meridionals (Å)**	
4.73	4.72
**Off Meridionals**	
4.32	4.52
	4.18
	3.9
**Equatorials (Å)**	
21	22
18.2	16
14.7	12.4
11.3	11
9.77	9.21
6.26	8.10
5.78	6.74
4.99	5.9
	5.6
	5.4

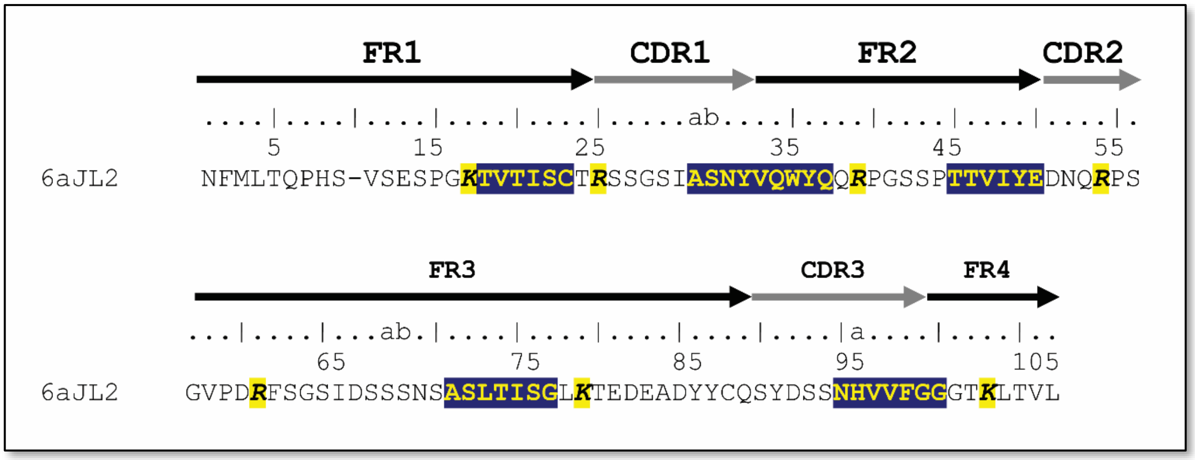

Figure S7. Relative position of the Arg/Lys residues respecting to the regions of the protein 6aJL2 predicted as aggregation-prone/fibrillogenic by the web-based tool AmylPred2 (http://biophysics.biol.uoa.gr/AMYLPRED2). The sequence of 6aJL2 protein is shown in one-letter code. The residues Arg (R) and Lys (K) are shown in bold, italic, and shadowed in yellow. The aggregation-prone/fibrillogenic regions are shown in yellow letter shadowed in dark blue.

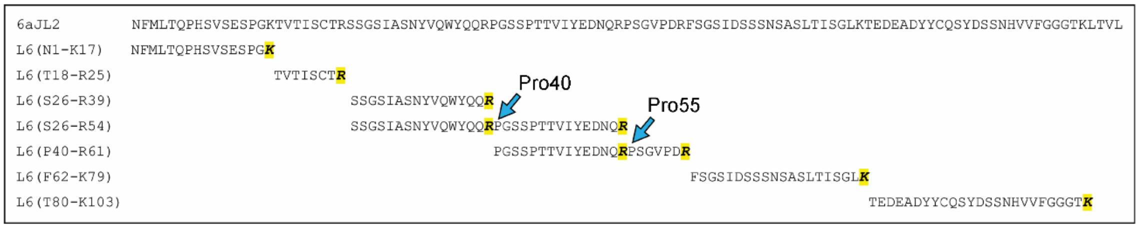

Figure S8. Sequence of the synthetic peptides that were assayed to unambiguously establishing the capability of each of the tryptic fragments of 6aJL2 to form amyloid-like aggregates. The sequence of 6aJL2 protein, in one-letter code, is shown at the top of the figure. The name used for identifying each peptide is shown at the left corner. Arg (R) and Lys (K) residues, representing the site of cleavage by trypsin, are shown in bold, italic and shadowed in yellow. Pro40 and Pro55 are indicated by arrows.

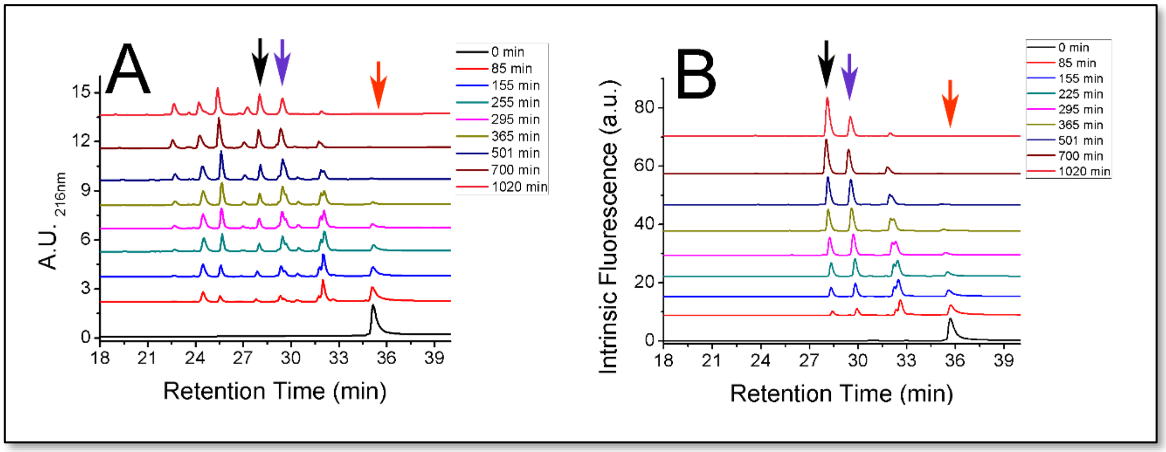

Figure S9. Analytical reversed-phase high performance liquid chromatography of aliquots withdrawn at different time of incubation of a 3 mg/ml solution of protein 6aJL2 in 50 mM TrisHCl buffer pH 8.0 with trypsin (as shown in the legend). Trypsin was added to a protein/trypsin w:w ratio of 200:1 and the sample was incubated at 37°C without agitation. The chromatography analysis was performed in a Vydac 218TP53 C18 analytical column (GRACE, Columbia, MD 21044, USA). The elution of the sample components was accomplished with a gradient of 0-60% of acetonitrile with 0.1% (v/v) TFA (solvent B) in miliQ-grade water with 0.1% (v/v) TFA (solvent A) in 60 min, keeping the flow at 0.5 ml/min. The data represented are the time-dependent variation of ***A)*** the absorbance at a wavelength of 216 nm and ***B)*** intrinsic fluorescence at 350 nm, exciting the sample at 295 nm. The arrows coloured black, blue and red point the fractions containing the peptide $_{26}$SSGSIASNYVQ***W***YQQR$_{39}$, $_{26}$SSGSIASNYVQ***W***YQQ**R**PGSSPTTVIYEDNQR$_{54}$, and the intact protein, respectively. The two first components are generated by cleavage of the protein 6aJL2 at positions Arg39 and Arg54, respectively, which are followed in the C-terminal site by a Pro residue. Both fragments appear relatively early during the proteolysis, indicating that proteolysis in both sites is not severely hindered by the presence of Pro. Note that both proteolytic fragments contain the only Trp residue of the protein (Trp35), which is the main source of intrinsic fluorescence.

Table S10. Sequence of the 16-mer synthetic peptides with point-mutation to Pro used in the Pro-scanning mutagenesis analysis of peptide Ser26-Arg39.

Peptide Name	Peptide sequence $NH_2 \rightarrow COOH$ (one letter code)
L6-Wt	$_{26}$SSGSIASNYVQWYQQR$_{39}$
L6-R39P	$_{26}$SSGSIASNYVQWYQQ**P**$_{39}$
L6-Q38P	$_{26}$SSGSIASNYVQWYQ**P**R$_{39}$
L6-Q37P	$_{26}$SSGSIASNYVQWY**P**QR$_{39}$
L6-Y36P	$_{26}$SSGSIASNYVQW**P**QQR$_{39}$
L6-W35P	$_{26}$SSGSIASNYVQ**P**YQQR$_{39}$
L6-Q34P	$_{26}$SSGSIASNYV**P**WYQQR$_{39}$
L6-V33P	$_{26}$SSGSIASNY**P**QWYQQR$_{39}$
L6-Y32P	$_{26}$SSGSIASN**P**VQWYQQR$_{39}$
L6-N31P	$_{26}$SSGSIAS**P**YVQWYQQR$_{39}$
L6-S30bP*	$_{26}$SSGSIA**P**NYVQWYQQR$_{39}$
L6-A30aP*	$_{26}$SSGSI**P**SNYVQWYQQR$_{39}$
L6-I30P	$_{26}$SSGS**P**ASNYVQWYQQR$_{39}$
L6-S29P	$_{26}$SSG**P**IASNYVQWYQQR$_{39}$
L6-G28P	$_{26}$SS**P**SIASNYVQWYQQR$_{39}$
L6-S27P	$_{26}$S**P**GSIASNYVQWYQQR$_{39}$
L6-S26P	$_{26}$**P**SGSIASNYVQWYQQR$_{39}$

* According to the numbering system of Chothia and Lesk[5].

Table S11. Synthetic peptide library evaluated for determining the aggregation propensity of the region encompassing the positions from 26 to 39 in the immunoglobulin light chains of both types, κ and λ. The library is composed of 26 synthetic peptides and represent the sequences encoded by 35 V_L gene segments, 12 λ and 23 κ.

Peptide name[1] (V_L gene segment)[2]	Peptide sequence[3]/Length in amino acids	AL-Base n[4] / %[5]	*In vitro* fibrillogenesis[6]
λ1-1a (*IGLV1-36*)	$_{26}$SSSNIGNNAVNWYQQL$_{39}$ / 16 aa	5/0.8	Yes
λ1-1c (*IGLV1-44*)	$_{26}$SSSNIGSNTVNWYQQL$_{39}$ / 16 aa	80/13	Yes
λ1-1e (*IGLV1-40*)	$_{26}$SSSNIGAGYDVHWYQQL$_{39}$ / 17 aa	11/1.8	No
λ2-2a2 (*IGLV2-14*)	$_{26}$TSSDVGGYNYVSWYQQH$_{39}$ / 17 aa	59/9.6	Yes
λ2-2d (*IGLV2-18*)	$_{26}$TSSDVGSYNRVSWYQQP$_{39}$ / 17 aa	0	No
λ2-2b2 (*IGLV2-23*)	$_{26}$TSSDVGSYNLVSWYQQH$_{39}$ / 17 aa	14/2.3	Yes
λ3-3r (*IGLV3-1*)	$_{26}$DKLGDKYACWYQQK$_{39}$ / 14 aa	163/26.5	No
λ3-3l (*IGLV3-19*)	$_{26}$DSLRSYYASWYQQK$_{39}$ / 14 aa	13/2.1	Yes?
λ3-3h (*IGLV3-21*)	$_{26}$DALPKQYAYWYQQK$_{39}$ / 14 aa	21/3.4	No
λ3-V2-19 (*IGLV3-27*)	$_{26}$DVLAKKYARWFQQK$_{39}$ / 14 aa	2/0.3	No
λ3-3e (*IGLV3-22*)	$_{26}$DVLGENYADWYQQK$_{39}$ / 14 aa	0	No
λ6-6a (*IGLV6-57*)	$_{26}$SSGSIASNYVQWYQQR$_{39}$ / 16 aa	113/18.3	Yes

κ1-O8/O18 (*IGKV1D-33 / IGKV1-33*)	$_{26}$SQDISNYLNWYQQK$_{39}$ / 14 aa	50/13<br>(n=63)/(32.8)	Yes
κ1-O2/O12 (*IGKV1D-39* / *IGKV1-39*)	$_{26}$SQSISSYLNWYQQK$_{39}$ / 14 aa	19/1<br>(n=20)/10.4	Yes
κ1-L1 (*IGKV1-16*)	$_{26}$SQGISNYLAWFQQK$_{39}$ / 14 aa	17/8.9	Yes
κ1-L14 (*IGKV1D-17*)	$_{26}$RQGISNYLAWFQQK$_{39}$ / 14 aa	0	Yes
κ1-L12 (*IGKV1-5*)	$_{26}$SQSISSWLAWYQQK$_{39}$ / 14 aa	31/16.1	
κ1-L15/L5/L19<br>(*IGKV1D-16* / *IGKV1-12* / *IGKV1D-12*)	$_{26}$SQGISSWLAWYQQK$_{39}$ / 14 aa	1/5/2<br>(n=8)/4.2	Yes
κ1-A30/L11 (*IGKV1-17* / *IGKV1-6*)	$_{26}$SQGIRNDLGWYQQK$_{39}$ / 14 aa	0	No
κ1-L4 (*IGKV1-13*)	$_{26}$SQGISSALAWYQQK$_{39}$ / 14 aa	0	Yes
κ1-L8/L23/L24<br>(*IGKV1-9* / *IGKV1D-43* / *IGKV1D-8*)	$_{26}$SQGISSYLAWYQQK$_{39}$ / 14 aa	0	Yes
κ2-O1/O11 (*IGKV2D-40* / *IGKV2-40*)	$_{26}$SQSLLDSDDGNTYLDWYLQK$_{39}$ / 20 aa	0	No
κ2-A3/A19 (*IGKV2D-28* / *IGKV2-28*)	$_{26}$SQSLLHSNGYNYLDWYLQK$_{39}$ / 19 aa	2/0	No

		(n=2)/1	
κ3-L6 (*IGKV3-11*)	$_{26}$SQSVSSYLAWYQQK$_{39}$ / 14 aa	0	Yes
κ3-L2 (*IGKV3-15*)	$_{26}$SQSVSSNLAWYQQK$_{39}$ / 14 aa	4/2.1	Yes
κ4-B3 (*IGKV4-1*)	$_{26}$SQSVLYSSNNKNYLAWYQQK$_{39}$ / 20 aa	41/21.4	Yes
κ4-B3 (*IGKV4-1/* O1/O11)[7]	$_{26}$SQSVL***D***S***DD***NKNYL***D***WYQQK$_{39}$/ 20 aa	-	No

[1] The name of each peptide includes the isotype, κ or λ, to which it belongs and the V-BASE identification name of the germline V_L gene segment encoding it, which is also used in AL-Base[6].

[2] It refers to the HUGO Gene Nomenclature Committee[7] approved symbol of the V_L gene segment encoding the sequence of the peptide. Note that some phylogenetically closely related V_L gene segments, as *IGKV1D-16*, *IGKV1-12*, and *IGKV1D-12*; all member of the V_L subgroup κ1, encode the same amino acid sequence for the positions 26 to 36 of the V_L domain. In these cases, their respective symbols are shown separated by slash.

[3] The peptide sequence, starting at position 26 and ending at position 39, is shown using the one-letter code.

[4] It refers to the number of sequences of AL deposited at the AL-Base[6] that is encoded by the germline V_L gene segment. In the case that the sequence of the peptide is encoded by more than one V_L gene segment, the data of each gene is shown separated by a slash and the sum (n) is shown between parenthesis.

[5] It refers to the percentage represented by the number of AL sequences derived from the germline V_L gene segment(s) in the total of AL sequences of a specific isotype (κ or λ) deposited in the AL-Base. At the time of the analysis (October 2018), there were 808 AL sequences compiled at the AL-Base, 616 λ and 192 κ.

[6] It refers to the result of the *in vitro* fibrillogenesis assay performed as described in Methods and shown in figures 10 and S12.

[7] This peptide has the sequence encoded by the κ4 gene segment B3 (*IGKV4-1)*, but with replacement to Asp at the same positions where this residue is present in peptide κ2-O1/O11, which is also 20 residues in length.

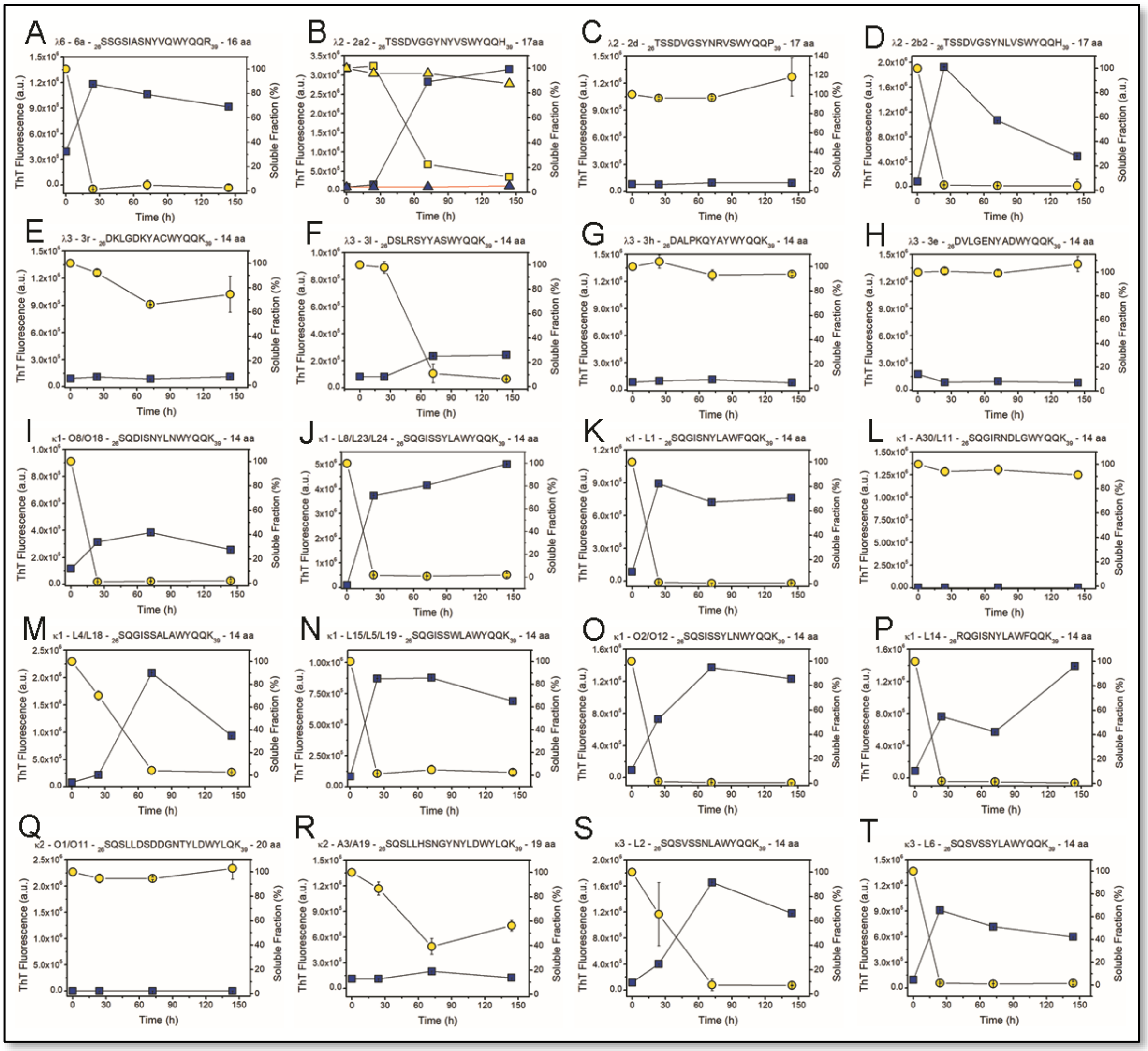

Figure S12. *In vitro* fibrillogenesis of twenty of the twenty-five κ and λ synthetic peptides composing the Ser26-Arg39-homologous peptide library. The data shown are the average of the samples duplicate plus the 95% confidence interval of the ThT fluorescence (blue square) and the soluble fraction (yellow circle). The soluble fraction was calculated from the value of intrinsic fluorescence of the supernatant obtained by centrifugation of an aliquot taken from the sample, as described in Methods. The name, sequence in one-letter code and number of amino acid residues of each peptide is shown at the top of the graph (see Table S11). Note that some κ peptides are encoded by more than one V_L gene segment. For example, peptide κ1-L15/L5/L19 is encoded by the V_L gene segments L15, L5, and L19, all member of the V_L subgroup κ1.

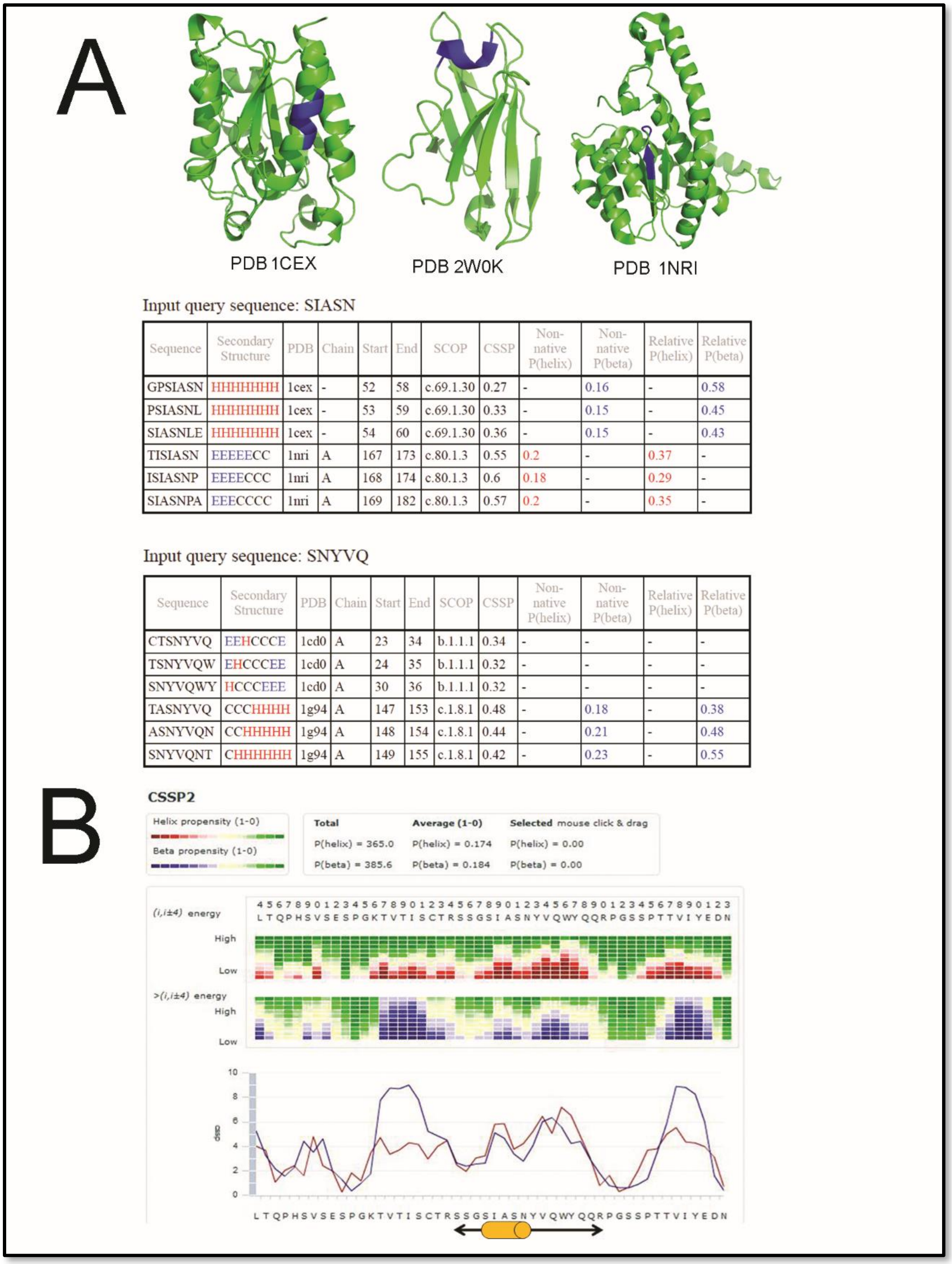

Sequence	Secondary Structure	PDB	Chain	Start	End	SCOP	CSSP	Non-native P(helix)	Non-native P(beta)	Relative P(helix)	Relative P(beta)
GPSIASN	HHHHHHH	1cex	-	52	58	c.69.1.30	0.27	-	0.16	-	0.58
PSIASNL	HHHHHHH	1cex	-	53	59	c.69.1.30	0.33	-	0.15	-	0.45
SIASNLE	HHHHHHH	1cex	-	54	60	c.69.1.30	0.36	-	0.15	-	0.43
TISIASN	EEEEECC	1nri	A	167	173	c.80.1.3	0.55	0.2	-	0.37	-
ISIASNP	EEEECCC	1nri	A	168	174	c.80.1.3	0.6	0.18	-	0.29	-
SIASNPA	EEECCCC	1nri	A	169	182	c.80.1.3	0.57	0.2	-	0.35	-

Sequence	Secondary Structure	PDB	Chain	Start	End	SCOP	CSSP	Non-native P(helix)	Non-native P(beta)	Relative P(helix)	Relative P(beta)
CTSNYVQ	EEHCCCE	1cd0	A	23	34	b.1.1.1	0.34	-	-	-	-
TSNYVQW	EHCCCEE	1cd0	A	24	35	b.1.1.1	0.32	-	-	-	-
SNYVQWY	HCCCEEE	1cd0	A	30	36	b.1.1.1	0.32	-	-	-	-
TASNYVQ	CCCHHHH	1g94	A	147	153	c.1.8.1	0.48	-	0.18	-	0.38
ASNYVQN	CCHHHHH	1g94	A	148	154	c.1.8.1	0.44	-	0.21	-	0.48
SNYVQNT	CHHHHHH	1g94	A	149	155	c.1.8.1	0.42	-	0.23	-	0.55

Figure S13. A) Search of chameleon sequences in 6aJL2 protein performed with the web-based computational tool Net-CSSP[8]. The analysis shows that the segments $_{29}$SIASN$_{31}$ and $_{30b}$SNYVQ$_{34}$, both of five residues in length, are found in both helical and beta context in

several native proteins. The images at the top show the location (highlighted in blue) of the sequence $_{29}$SIASN$_{31}$ in the crystallographic structure of proteins cutinase from *Fusarium solani* (PDB 1CEX), V_L 6aJL2 (PDB 2W0K) and putative Phosphosugar Isomerase HI0754 from *Haemophilus influenzae* (PDB 1NRI). ***B)*** Prediction of contact-dependent secondary structure propensity performed on 6aJL2 protein with the web-based computational tool CSSP2 (dual network)[9]. In the bottom graphic, the blue and red lines represent the propensity to adopt β-sheet and α-helix, respectively. Only the data of the protein segment from Leu4 to Ans52 is shown. The double-headed arrow indicates the position of the highly fibrillogenic segment Ser26-Arg39. Note that the algorithm predicts that this segment displays similar propensity to adopt α helix and β sheet conformation. The yellow cylinder superimposed on the two-headed arrow indicates the helical segment spanning the SIASN sequence of the CDR1 in the native 6aJL2 protein.

A

Peptide	Amyloid Fibrils	Sequence	Length (aa)
λ6-6a	(Yes)	SSGSIAS--NYVQWYQQR	16
λ1-1a	(Yes)	..SN.GN--.A.N....L	16
λ1-1c	(Yes)	..SN.G.--.T.N....L	16
λ1-1e	(No)	..SN.GA***G***Y***D***-.H....L	17
λ2-2a2	(Yes)	T.SDVGG-Y...S....H	17
λ2-2d	(No)	T.SDVG.-Y.***R***.S....P	17
λ2-2b2	(Yes)	T.SDVG.-Y.L.S....H	17
λ3-3r	(No)	DK–LG***D***---***K***.AC....K	14
λ3-31	(Yes?)	D.-L***R***.---Y.AS....K	14
λ3-3h	(No)	DA-L***PK***---Q.AY....K	14
λ3-V2-19	(No)	DV-L.***K***---***K***.A***R***.F..K	14
λ3-3e	(No)	DV-LG***E***---..A***D***....K	14

B

Peptide	Amyloid Fibrils	Sequence	Length (aa)
κ1-L8/L23/L24	(Yes)	SQGISS------YLAWYQQK	14
κ1-O8/O18	(Yes)	..D..N------..N.....	14
κ1-02/012	(Yes)	..S...------..N.....	14
κ1-L1	(Yes)	.....N------....F...	14
κ1-L14	(Yes)	R....N------....F...	14
κ1-L15/L5/L19	(Yes)	......------W.......	14
κ1-A30/L11	(No)	....***R***N------***D***.G.....	14
κ1-L4/18	(Yes)	......------A.......	14
κ2-O1/O11	(No)	..SLL***D***S***DD***GNT..***D***..L..	20
κ2-A3/A19	(No)	..SLLHSN-GYN..***D***..L..	19
κ3-L6	(Yes)	..SV..------........	14
κ3-L2	(Yes)	..SV..------N.......	14
κ4-B3	(Yes)	..SVLYSSNNKN........	20

Figure S14. Sequence alignment and amyloid-forming capacity of the synthetic peptides of A) λ and B) κ type conforming the Ser26-Arg39-homologous peptide library. The peptides, shown in the one-letter code, were aligned with Clustal X[10] multiple alignment software, taking as reference the sequence at the top of each column. Only the residues different to that occupying the position in the peptide of reference are shown. The short dashes represent gaps introduced by the alignment algorithm. The amyloid-forming capacity was evaluated by incubating the peptides diluted in PBS pH 7.4 plus GdnHCl 1.0 M at 37°C with constant orbital agitation. The presence of amyloid was determined by the ThT fluorescence assay[11] and TEM analysis. Acid (Asp and Glu) and basic (Arg and Lys) residues, as well as

Pro, at positions potentially important for peptide aggregation are shown in bold and italic and highlighted in yellow.

Supplementary text S15.

1. Fibrillogenesis assay of the wild-type synthetic peptide S26-R39 at increasing concentrations of GdnHCl.

Aim

Establish the dependence between the concentration of GdnHCl and the ability of the wild-type peptide Ser26-Arg39 to aggregate as amyloid.

Experimental design

- Approximately, 2 mg of dried 96% pure synthetic peptide Ser26-Arg39 (GenScript Biotech Corp, Piscataway, NJ, USA) were dissolved in 0.7 ml of 6.0 M GdnHCl solution. The solution was incubated at 95°C, without agitation, for 30 minutes.
- The solution was centrifuge at 25,000 g for 30 minutes at room temperature to pellet any persisting aggregate. Then, the supernatant was carefully recovered, avoiding dragging the sediment, and transferred to another tube.
- An aliquot (50 µl) was withdrawn from the peptide solution and mixed with 600 µl of a 6.0 M GdnHCl solution. The absorbance at 280 nm (Ab_{280}) of the dilution was determined in a spectrophotometer and the concentration of the peptide was calculate using the following equation: $[mg/ml] = (Ab_{280} \times D)/(8480/1874)$

Where D is the dilution (x13), 8480 and 1870 are the molar extinction coefficient and the MW of the peptide, respectively, calculated from the sequence by the computational tool ProtParam (http://web.expasy.org/cgi-bin/protparam).

- Fourteen aliquots (300 µl) containing 150 µM of the peptide and increasing concentration of GndHCl (1.0 M, 1.25 M, 1.5 M, 1.7 M, 1.9 M, 2.1 M, 2.2 M, 2.35 M, 2.5 M, 2.75 M, 3.0 M, 4.0 M, 5.0 M, and 6.0 M) were prepared and placed in 2 ml polypropylene microcentrifuge tubes (AXYGEN, Corning, NY, USA, Cat. No. MCT-200-C). The samples were incubated at 37°C with constant agitation at 500 r.p.m. in an orbital shaker Thermomixer Comfort (EPPENDORF AG, Hamburg, Germany).
- At time T=0, and after 16 and 40 hours of incubation, an aliquot (20 µl) of each sample was taken and mixed with 1.0 ml of 20 µM ThT solution. The ThT fluorescence was registered from 460 nm to 510 nm, exciting the sample at 450 nm.

- At the same time, a second aliquot (50 μl) was withdrawn from the sample and centrifuged at 25,000 g for 30 minutes at room temperature. Then, an aliquot (20 ul) of the supernatant was taken and mixed with 1.0 ml of PBS pH 7.4. The intrinsic fluorescence of the solution was registered from 300 nm to 410 nm, exciting the sample at 280 nm.

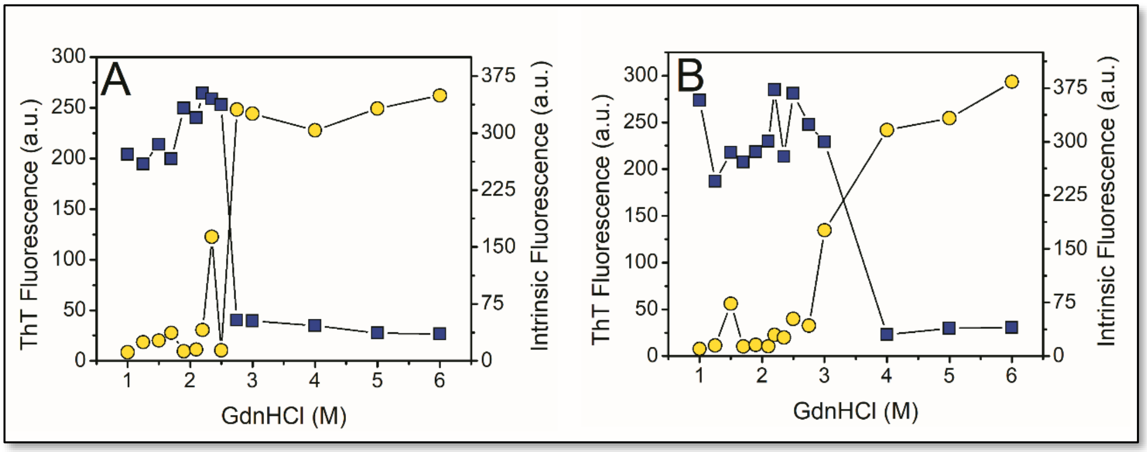

Figure S16. Fibrillogenesis of the wild-type peptide Ser26-Arg39 at increasing concentration of guanidine hydrochloride (GdnHCl). The data represented are the ThT fluorescence (blue square) and intrinsic fluorescence of the supernatant (yellow circle) of the sample after ***A)*** sixteen and ***B)*** forty hours of incubation. The ThT fluorescence and intrinsic fluorescence of the supernatant were performed as described in Supplementary text S15.

REFERENCES

1 del Pozo-Yauner, L., Becerril-Luján, B., Ochoa-Leyva, A., Rodriguez-Ambriz, S.L., Perez Carrion, J.I., Zavala-Padilla, G., Sanchez-Lopez, R., Fernandez Velasco, D.A. . in *Physical Biology of Proteins and Peptides* (ed Luis. Olivares-Quiroz, Guzmán-López, Orlando., Jardón-Valadez, Hector Eduardo.) Ch. The Structural Determinants of the Immunoglobulin Light Chain Amyloid Aggregation, 1-28 (Springer, 2015).

2 Goldschmidt, L., Teng, P. K., Riek, R. & Eisenberg, D. Identifying the amylome, proteins capable of forming amyloid-like fibrils. *Proc Natl Acad Sci U S A* **107**, 3487-3492, doi:10.1073/pnas.0915166107 (2010).

3 Thompson, M. J. *et al.* The 3D profile method for identifying fibril-forming segments of proteins. *Proc Natl Acad Sci U S A* **103**, 4074-4078, doi:10.1073/pnas.0511295103 (2006).

4 Kuhlman, B. & Baker, D. Native protein sequences are close to optimal for their structures. *Proc Natl Acad Sci U S A* **97**, 10383-10388 (2000).

5 Al-Lazikani, B., Lesk, A. M. & Chothia, C. Standard conformations for the canonical structures of immunoglobulins. *J Mol Biol* **273**, 927-948, doi:10.1006/jmbi.1997.1354 (1997).

6 Bodi, K. *et al.* AL-Base: a visual platform analysis tool for the study of amyloidogenic immunoglobulin light chain sequences. *Amyloid : the international journal of experimental and clinical investigation : the official journal of the International Society of Amyloidosis* **16**, 1-8, doi:10.1080/13506120802676781 (2009).

7 White, J. A. *et al.* Guidelines for human gene nomenclature (1997). HUGO Nomenclature Committee. *Genomics* **45**, 468-471 (1997).

8 Kim, C., Choi, J., Lee, S. J., Welsh, W. J. & Yoon, S. NetCSSP: web application for predicting chameleon sequences and amyloid fibril formation. *Nucleic Acids Res* **37**, W469-473, doi:10.1093/nar/gkp351 (2009).

9 Yoon, S., Welsh, W. J., Jung, H. & Yoo, Y. D. CSSP2: an improved method for predicting contact-dependent secondary structure propensity. *Comput Biol Chem* **31**, 373-377, doi:10.1016/j.compbiolchem.2007.06.002 (2007).

10 Larkin, M. A. *et al.* Clustal W and Clustal X version 2.0. *Bioinformatics* **23**, 2947-2948, doi:10.1093/bioinformatics/btm404 (2007).

11 Naiki, H., Higuchi, K., Hosokawa, M. & Takeda, T. Fluorometric determination of amyloid fibrils in vitro using the fluorescent dye, thioflavin T1. *Anal Biochem* **177**, 244-249 (1989).
